# Genetic Patterns Found in the Nuclear Localization Signals (NLSs) Associated with EBV-1 and EBV-2 Provide New Insights into Their Contribution to Different Cell-Type Specificities

**DOI:** 10.3390/cancers13112569

**Published:** 2021-05-24

**Authors:** Louise Zanella, María Elena Reyes, Ismael Riquelme, Michel Abanto, Daniela León, Tamara Viscarra, Carmen Ili, Priscilla Brebi

**Affiliations:** 1 Laboratory of Integrative Biology (LIBi), Scientific and Technological Bioresource Nucleus-Center for Excellence in Translational Medicine (BIOREN-CEMT), Universidad de La Frontera, Temuco 4810296, Chile; zanella.bio@gmail.com (L.Z.); m.reyes14@ufromail.cl (M.E.R.); or d.leon02@ufromail.cl (D.L.); tamara.viscarra@ufrontera.cl (T.V.); carmen.ili@ufrontera.cl (C.I.); 2 Departamento de Ciencias Básicas, Facultad de Ciencias, Universidad Santo Tomas, Santiago 8370003, Chile; 3 Instituto de Ciencias Biomédicas, Facultad de Ciencias de la Salud, Universidad Autónoma de Chile, Temuco 4810101, Chile; ismael.riquelme@uautonoma.cl; 4 Scientific and Technological Bioresource Nucleus (BIOREN), Universidad de La Frontera, Temuco 4811230, Chile; michel.abanto@ufrontera.cl

**Keywords:** Epstein–Barr virus (EBV), EBV nuclear antigen EBNA 3A (EBNA3A), recombination, phylogeny, EBV classification, nuclear localization signal (NLS)

## Abstract

**Simple Summary:**

The Epstein–Barr virus (EBV) has been implicated in several human neoplastic diseases. The EBV-1 can transform B cells into LCL more efficiently than EBV-2, and EBV-2 preferentially infects T-cell lymphocytes. The EBNA3A oncoprotein has an essential role in B-cell transformation. The six peptide motifs called nuclear localization signals (NLSs) from EBNA3A ensure nucleocytoplasmic protein trafficking. Multiple NLSs have been suggested to enhance EBNA3 function or different specificities to different cell types; however, a comprehensive assessment of their genetic variability has not been addressed. Our objective was to study the NLSs’ variability and their relationship with EBV types. Based on a comprehensive analysis of over a thousand EBNA3A sequences from different clinical manifestations and geographic locations, we found that EBNA3A from EBV-2 has two of the six NLSs altered, and genetic patterns in the NLSs are associated with EBV-1 and EBV-2.

**Abstract:**

The Epstein–Barr virus (EBV) is a globally dispersed pathogen involved in several human cancers of B-cell and non-B-cell origin. EBV has been classified into EBV-1 and EBV-2, which have differences in their transformative ability. EBV-1 can transform B-cells into LCL more efficiently than EBV-2, and EBV-2 preferentially infects T-cell lymphocytes. The EBNA3A oncoprotein is a transcriptional regulator of virus and host cell genes, and is required in order to transform B-cells. EBNA3A has six peptide motifs called nuclear localization signals (NLSs) that ensure nucleocytoplasmic protein trafficking. The presence of multiple NLSs has been suggested to enhance EBNA3 function or different specificities in different cell types. However, studies about the NLS variability associated with EBV types are scarce. Based on a systematic sequence analysis considering more than a thousand EBNA3A sequences of EBV from different human clinical manifestations and geographic locations, we found differences in NLSs’ nucleotide structures among EBV types. Compared with the EBNA3A EBV-1, EBNA3A EBV-2 has two of the six NLSs altered, and these mutations were possibly acquired by recombination. These genetic patterns in the NLSs associated with EBV-1 and EBV-2 provide new information about the traits of EBNA3A in EBV biology.

## 1. Introduction

The Epstein–Barr Virus (EBV) is a human herpesvirus that belongs to the family *Herpesviridae*. EBV is related to the development of infectious mononucleosis (IM) [1], as well as potentially fatal neoplastic diseases, such as Burkitt lymphoma (BL) [2], nasopharyngeal cancer (NPC) [3], and gastric cancer (GC) [4]. The viral genome consists of about 170 kb double-stranded DNA coding over 100 proteins [5], including the nuclear antigens (EBNA)—EBNA-1, -2, -3A, -3B -3C, and leader protein (EBNA-LP)—and latent membrane proteins LMP-1 and -2 [6]. EBV is classified into EBV type 1 (EBV-1) and type 2 (EBV-2), based on specific polymorphisms of the *EBNA2* and *EBNA3A*, *3B*, and *3C* genes [7,8]. The two types have functional differences in their ability to transform human B cells. EBV-1 induces the continuous proliferation (known as “transformation”) of human B cells more efficiently than EBV-2 in vitro [8,9]. Although EBV-2 has been associated with lower efficiency in immortalizing B lymphocytes in vitro, it has been detected in BL tumors [10]. Unlike EBV-1, which has a tropism for B lymphocytes, EBV-2 preferentially infects T-cells in cell culture [11].

Among latency proteins, EBNA3 proteins are transcription regulators of virus and host cell genes [12,13]. The EBNA3 family consists of three proteins (EBNA3A, EBNA3B, and EBNA3C) that possibly originated from the triplication of a single ancestral gene [14]. The *EBNA3* genes are expressed via polycistronic transcription, and share a characteristic exon structure arranged in tandem: a short 5′ exon and a long 3′ exon, separated by a short intron [15].

EBNA3A comprises 944 amino acids, and contains multiple sequence-specific interaction regions for various proteins [16]. To regulate cellular gene expression, EBNA3A interacts with the cell’s DNA-binding protein (RBP-JK) and with the carboxyl-terminal-binding protein (CtBP) [16,17]. These proteins play an essential role in reprogramming the cellular gene transcription process to transform the B lymphocyte, contributing to the development of malignancies [18].

The EBNA3 family proteins are transported from the cytoplasm to the nucleus by the signal-mediated process. Nucleocytoplasmic trafficking occurs based on the peptide motif called nuclear localization signal (NLS) tags. The nuclear pore complexes (NPC) recognize these short, positively charged motifs and actively import EBNA3A to the nucleus [19,20]. EBNA3A has multiple (six) NLSs that ensure protein transport to the nucleus. Examining the NLSs via partial EBNA3A deletion constructs indicates that simultaneous mutations in NLS3 and NLS4 result in partial EBNA3A-mutated proteins restricted to the cell’s cytoplasm [21].

The classic EBV classification was recently revisited, updating the recombination impact on the EBV genome and demonstrating the consistent presence of sites of recombination in the *EBNA3A* gene [22]. However, an evaluation of the effect of recombination in the *EBNA3A* gene or its NLS regions has been not addressed.

Although some alterations to the peptide motif of EBNA3A NLSs have already been demonstrated, this was only in the context of partial proteins [21]. To date, these changes have not been observed in sequences recovered from natural human EBV infection, and moreover, there is a scarcity of EBV sequences from South America. In the present study, we report the first 12 EBNA3A sequences of gastric cancer bulk from Chile: 3 from EBV type 2 (the first non-Asian sequences) and 9 from EBV type 1, doubling the number of EBV EBNA3A sequences from South America. Moreover, we investigated the genetic diversity and delineated the effect of recombination in the *EBNA3A* gene structure.

Our results showed that *EBNA3A* from EBV-2 has two (NLS3 and NLS4) of the six NLSs altered (non-canonical), and that these non-synonymous mutations were possibly acquired by recombination. We found that the genetic patterns in the NLSs are associated with EBV-1 and EBV-2, and we suggested that this could be contributing to different cell-type specificities.

## 2. Materials and Methods

### 2.1. Fresh-Frozen Gastric Cancer Tissues for PCR Analysis

Gastric biopsy samples were obtained from 54 adult patients, of both sexes, after surgical resection in the Hernán Henríquez Aravena Hospital. The samples were collected with ethics approval (Universidad de La Frontera N° 20/011) between 2008 and 2012.

### 2.2. DNA Extraction and PCR Assay

DNA was isolated from the fresh-frozen GC tissues using the EZNA Tissue DNA Kit (Omega Bio-Tek, Norcross, USA, GA) according to the manufacturer’s instructions. EBV screening was performed by PCR amplification of the *EBNA3A* region. We thus generated 3 overlapping fragments spanning the entire *EBNA3A* region for the 12 EBV positive samples. The sequences of the primers were as follows: 5′ region (primers EBNA3A-20F–GTGCGGTGTTGGTGAGTCACACT and EBNA3A1392R–CGCCGACCCGTGACTGGTA, ~1430 pb fragment), center region (primers EBNA3A1041F–CACAKGCTTGGAATGCCGGCTT and EBNA3A2155R–GCCCATACTAGCCCKYAACG, ~1115 pb fragment), and 3′ region (primers EBNA3A2136F–CGTTRMGGGCTAGTATGGGC and EBNA3A + 33R–CGGGCGTATTATCAGTGGGT, ~820 pb fragment). The PCR reactions were carried out under the following conditions: 98 °C for 3 min, followed by 35 cycles at 95 °C for 30 s, 55 °C for 30 s, and 72 °C for 1 min. The amplified *EBNA3A* fragments were sequenced using Macrogen’s Sanger sequencing system (https://dna.macrogen.com/, accessed on 21 March 2018). The 12 *EBNA3A* sequences of gastric cancer bulk from Chile were deposited in GenBank under accession numbers MW219146–MW219157.

### 2.3. Sequence Dataset

One thousand and seventeen EBV genome sequences were obtained from Genbank (accessed on 8 April 2020). Sequence similarity searches with the Basic Local Alignment Search Tool (tBLASTn) were performed using EBNA3A amino acid queries P12977 and Q69138 as sequences equivalent to EBV-1 (the B95-8.Raji prototype) [23] (NC_007605) and EBV-2 (the AG876 [24] (NC_009334) prototype), respectively. Sequences lacking any part of the EBNA3 gene (e.g., AP019056, MH883771, LS992262) were eliminated from the analysis. A final dataset of 1006 sequences covering the entire *EBNA3A* gene (nucleotides 79955–82877 relative to the B95-8.Raji sequence including the exons and intron) was recovered from each genome, including *EBNA3A* sequences from the 12 samples sequenced in this study. The geographic origin, clinical manifestation, year of collection, and Genbank accession numbers are available in Supplementary Table S1.

### 2.4. Recombination and Phylogenetic Analyses

The recombination analysis of *EBNA3A* was performed using two different software programs: Gubbins, and RDP4. The recombination events were detected by super alignment output using Gubbins [25]. The maximum likelihood (ML) tree reconstruction was inferred using PhyML 3.0. (http://www.atgc-montpellier.fr/phyml/, accessed on 25 May 2020) under an HKY + Γ+I substitution model determined by SMS (http://www.atgc-montpellier.fr/sms/, accessed on 24 May 2020) based on the Akaike information criterion (AIC), and the reliability of the topology obtained was estimated with the approximate likelihood-ratio test (aLRT). The tree was visualized using Figtree v.1.4.3 (http://tree.bio.ed.ac.uk/software/figtree/, accessed on 8 May 2021) and results were treated with iTOL [26]. The recombination detection based on another approach was reexamined using the RDP4 phylogenetic method as well as the Chimaera [27], GENECONV [28], MaxChi [28], SisScan [29], 3Seq [30], and nucleotide substitution methods, all implemented in the RDP4 software package with default parameters (Supplementary Tables S2 and S3). When all algorithms had the threshold *p*-value of 0.05, the potential putative recombinant events were considered significant, using the Bonferroni correction.

## 3. Results

### 3.1. EBV-Associated Gastric Cancer from Chile

Fifty-four Chilean patients were recruited in our study. All patients with adenocarcinoma were diagnosed via surgery tumor resection and subsequent confirmation with histopathology. EBV was detected in 12 of the 54 (22%) gastric adenocarcinoma cases. The DNA from these samples was used to analyze variations in EBNA3A. Up to the present study, no EBV sequences from Chile were available. Here we reveal the first 12 EBV sequences of gastric cancer bulk from Chile (Supplementary Table S4).

### 3.2. EBNA3A Recovery from Genomes

Since no sequences of the *EBNA3A* gene were available in the databank, it was necessary to recover them from the EBV genomes. Members of the *EBNA3* family have two exons separated by a small intron [23]; therefore, complete *EBNA3A* CDS was recovered from EBV genomes through BLAST searches. In addition to these sequences, 12 Chilean *EBNA3A*s were sequenced in this study. Altogether, the dataset contains 1006 *EBNA3A* sequences representing the 5 continents (Supplementary Table S1). The clinical status of these infected individuals was diverse, from healthy to tumor carriers. The final dataset consists of 89 samples from America (Argentina, Brazil, Chile, and the USA), 93 from Europe (France, Germany, Poland, Ukraine, and the United Kingdom), 60 from Africa (Ghana, Kenya, Nigeria, North Africa, and Uganda), 32 from Oceania (Australia and Papua New Guinea), and 732 from Asia (China, Indonesia, Japan, Singapore, South Korea, Taiwan, and Vietnam). It was evident that there is an oversampling of Asian sequences (more than 70%).

### 3.3. Classifying EBV According to Traditional Classifications

**Figure 1:**
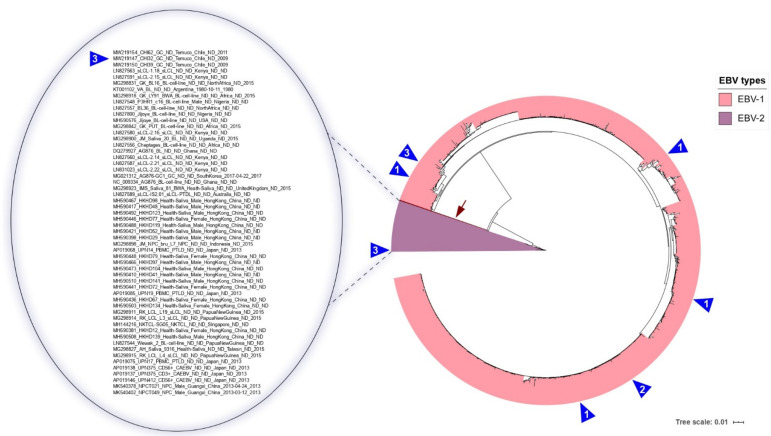
ML phylogenetic tree reconstruction of 1006 EBV *EBNA3A* sequences (2835 nt) from the 5 continents. In pink are the sequences classified as EBV-1; in lilac are the sequences classified as EBV-2. The two sequences (NPCT021 and NPCT049) closely related to the EBV-2 clade are in burgundy, and are depicted by a burgundy arrow. The zoom area shows in detail the EBV-2 sequences with their associated metadata. Blue triangles represent Chilean sequences identified in this study. The numbers inside the triangle indicate the number of sequences. The scale bar indicates nucleotide substitutions per site.

The standard criteria (type 1 and 2) for EBV were applied to classify the *EBNA3A* sequences (including the two exons and the small intron). The ML phylogenetic reconstruction showed that the main clade containing the EBV-1 prototype sequence (B95-8.Raji) congregated most EBV strains, reflecting the considerable number of EBV-1 sequences (Figure 1, tips highlighted in pink). The EBV-1 clade also contains nine Chilean sequences from this study: CHI29, CHI33, CHI34, CHI47, CHI53, CHI55, CHI66, CHI127, and CHI130 (Figure 1, tips indicated with blue triangles).

A minor clade was found to consist of ~5% of sequences (*n* = 56, see Figure 1, tips highlighted in lilac). This minor clade contains 13 sequences previously classified as belonging to the EBV-2 clade [22] (AG876–EBV-2 prototype, Cheptages, Jijoye, BL36, Wewak, sLCL-2.14, sLCL-2.16, sLCL1.18, sLCL2.21, sLCL-IS2.01, sLCL-2.15, sLCL-2.22, and AG876-GC1). The EBV-2 clade also contains three Chilean sequences from this study: CHI32, CHI39, and CHI62 (Figure 1, tips indicated with blue triangles), which shows the presence of EBV-2 in Chile. Two sequences (NPCT021 and NPCT049, Figure 1 tips highlighted in burgundy) were closely related to EBV-2 sequences.

The tree evidences the wide geographic distribution of EBV-1 compared with EBV-2. Half of the type 2 sequences came from the Asian continent (29/56), and the majority of the other half came from the African continent (16/56), with a meager number of samples from Oceania (5/56) and the Americas (5/56), and just one from the United Kingdom.

### 3.4. South American EBV Characteristics

To date, there have been 12 *EBNA3A* EBV sequences from South America: 6 from Argentina and 6 from Brazil, all representing individuals with BL. Except for the Argentinian sequence VA sampled in 1980, which belongs to EBV type 2, all other South American sequences belong to the EBV type 1 clade (Table S2). Due to the scarcity of EBV sequences from South America, we have revealed the first 12 *EBNA3A* sequences of EBV-positive gastric cancer from Chile. Therefore, this study doubles the number of EBV *EBNA3A* sequences from South America (Supplementary Table S4). Contrary to expectations, three of the Chilean sequences belong to the EBV type 2 clade, indicating that both types are circulating in Chile. In addition, this is the first non-Asian (MG021312 from South Korea) GC associated with EBV-2 sequences.

No unique signatures were found among the South American sequences. The *EBNA3A* sequence does not have sufficient signal to fine-tune discriminate subclades inside the EBV-1 group, but the phylogenetic analysis also revealed that the South American sequences belonging to the EBV-1 clade were widely dispersed in the tree, stressing the cosmopolitan nature of EBV type 1.

### 3.5. Two Non-Canonical NLSs Could Be Related to the Lower Efficiency of EBV-2 in Converting B Cells into Lymphoblastoid Cell Lines

The EBNA3A is essential for lymphocyte transformation, negatively affecting the transactivator EBNA-2 and the cell cycle [20]. The nucleus is the primary localization of this protein; therefore, changes in NLS residues could affect EBNA3A nucleocytoplasmic trafficking [20,21]. NLS3 and NLS4 have been reported previously as functional NLSs, and residue changes in these NLSs have been shown to affect EBNA3A partial protein transport to the nucleus [21].

**Figure 2:**
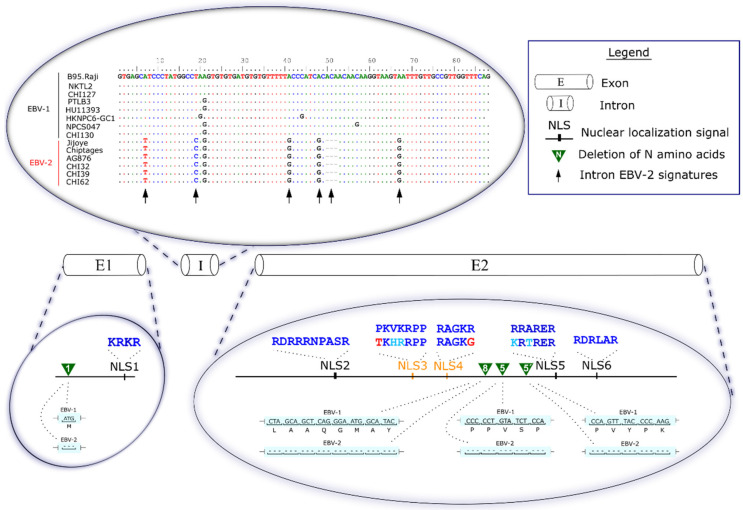
Schematic representation of EBNA3A amino acid architecture. Coding (exons E1 and E2) and non-coding (intron I) regions of EBNA3A are in the center of the image. The intron region’s upper zoom area shows the five unique nucleotides and three-nucleotide deletions (arrows) shared only with the EBV-2 sequences. At the bottom of the image are the EBNA3A nuclear location signals (NLSs), with the respective canonical amino acids of each of the six motifs in dark blue. Non-canonical NLSs with crucial changes are highlighted in orange (NLS3 and NLS4). The amino acid changes that would not directly affect the non-canonical motifs are in light blue. The amino acid changes that would directly affect the non-canonical motifs are highlighted in red. The green triangle indicates the four EBV-2-typical deletions identified in this study, and the amino acid deletions are highlighted with a light blue background. The numbers inside the triangles (1, 8, 5, and 5′) indicate the number of amino acids deleted. There were two deletions of five amino acids: the first deletion is indicated with the number 5, and the second with 5′.

Comparing the EBNA3A sequences made it possible to identify characteristic amino acid substitutions associated with EBV types (Figure 2). EBNA3A had six predicted NLSs [21], and we identified that six mutations that result in amino acid changes in the NLSs (NLS3, NLS4, and NLS5) present exclusively in EBV-2 strains (except for the NPCT021 and NPCT049 sequences from China, closely related to the EBV-2 clade). Among the six amino acid substitutions identified in the present study, two were non-conservative amino acid changes in NLS3 and NLS4 (Supplementary Table S5). P375T belongs to NLS number 3 (NLS3), and R398G belongs to NLS number 4 (NLS4) (see Figure 2, bottom). NLS3 (a pattern 7 NLS) had the characteristic proline in the first position substituted by a glycine. NLS4 had basic residue (arginine) substituted by an aliphatic (glycine). EBV-2 sequences present both two NLS motifs altered: NLS3 and NLS4, contrasting EBV-1 sequences with the six canonical NLSs.

### 3.6. Characteristic Deletions of EBV-2 in the EBNA3A Gene

The EBV-2 sequences show four particular regions with deletions in exonic regions (Figure 2, bottom; see Supplementary Table S6). (1) Deletion of one amino acid (Figure 2, the green triangle with the number 1 inside), M^15^, present in more than 96% of EBV-2 sequences (except for the NPCT021 and NPCT049 sequences from China, closely related to the EBV-2 clade). (2) Deletion of eight amino acids (Figure 2, the green triangle with the number 8 inside), ^451^LAAQGMAY^458^, present in more than 92% of EBV-2 sequences (except for the UPN375_CD3+ and UPN412_CD56+ sequences from Japan, and the NPCT021 and NPCT049 sequences from China). (3) First deletion of five amino acids (Figure 2, the green triangle with the number 5 inside), ^480^PPVSP^484^, present in more than 89% of EBV-2 sequences (except for UPN375_CD3+, UPN375_CD56+, and UPN412 from Japan, and NPCT021 and NPCT049 from China). (4) Second deletion of five amino acids (Figure 2, the green triangle with the number 5′ inside), ^548^PVYPK^552^, present in more than 96% of EBV-2 sequences (except for the NPCT021 and NPCT049 sequences from China).

Five EBV-2 sequences present unusual InDel patterns. (1) The UPN14 sequence from Japan has a deletion of 250 nt. (2) The VA sequence from Argentina has a deletion of 115 nt. (3 and 4) The UPN17 and UPN19 sequences from Japan have a deletion of 110 nt. These four sequences have relatively extensive deletions that include the deletion of eight amino acids and the first deletion of five amino acids identified in this study. Additionally, (5) the IMS_saliva_81 sequence from the United Kingdom has all deletions associated with EBV-2 (deletions 1,8, 5, and 5′), and immediately after the deletion of eight amino acids has a further deletion of 8 nt. The unusual InDel patterns could be representative of natural sequence variability, or could have originated from sequencing or assembly errors.

### 3.7. Unique Type 2 Signatures Retained in the Intron Region of EBNA3A

The *EBNA2* and *3A*, *3B*, and *3C* genes have a series of polymorphisms that allow the phylogenetic distinction of EBV-1 and EBV-2. We investigated such polymorphisms in the intron region of the *EBNA3A* coding region (88 nt, Figure 2 upper zoomed area). The alignment of the *EBNA3A* intron region revealed five EBV-2 signatures: A->T, T->C, A->G, A->G, and A->G, at the respective positions 80,368, 80,380, 80,402, 80,409, and 80,425. All nucleotide positions correspond to the EBV-2 prototype sequence AG876. In addition to the polymorphisms, a three-nucleotide deletion was also noted in all EBV-2 sequences. These polymorphisms and deletion patterns could be useful for further studies, or could serve as genetic markers for EBV classification.

### 3.8. EBNA3A Recombination Events Reside Exclusively in EBV-2

**Figure 3:**
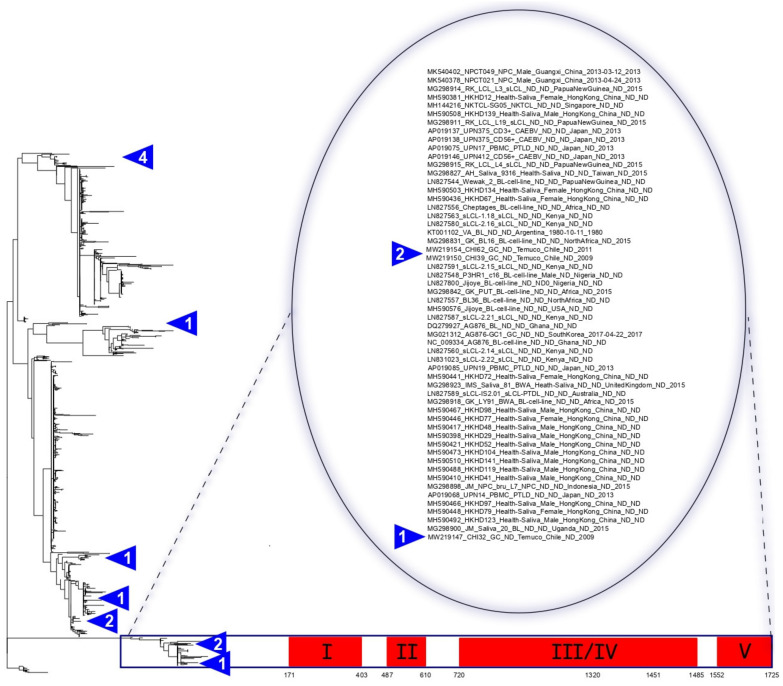
Phylogenetic tree and predicted recombination blocks in the *EBNA3A* gene. Phylogenetic tree reconstruction based on non-recombinant SNPs of 1006 EBV *EBNA3A* sequences on the Figure’s left side. The red blocks (I–V) indicate recombination areas common in multiple sequences that share descent at the bottom of the image. Below the red blocks are the positions corresponding to the *EBNA3A* nucleotide sequence. Sequences with predicted recombination blocks are in the zoomed area with their associated metadata. Only EBV-2 sequences had recombinant blocks predicted. Chilean sequences identified in this study are represented by blue triangles. The numbers inside the triangle indicate the number of sequences.

It was demonstrated that recombination is an important feature in the construction of the EBV genome architecture [6,22,31,32,33,34,35,36]. Based on this precedence, we identified five recombination blocks within more than a thousand sequences across the *EBNA3A* gene, based on SNP density (Figure 3 and Supplementary Table S7). Interestingly, only in EBV-2 sequences have recombination blocks been identified; conversely, no recombination blocks were identified in EBV-1 sequences. Three recombination blocks—recombinant block I (position 171–403 nt), recombinant block II (position 487–610 nt), and recombinant block V (position 1552–1725 nt)—were present in the same fifty-six sequences classified as EBV-2. One recombination block—the recombinant block III (position 720–1485 nt)—was present in all EBV-2 sequences except for the NPCT021and NPCT049 sequences; both of these sequences, from China, are closely related to the EBV-2 clade. One recombination block—recombinant block IV (position 1320–1451 nt)—was present in only four EBV sequences from Japan (UPN375_CD3+, UPN375_CD56+, UPN17, and UPN412). Interestingly, both non-canonical NLS changes identified in the present study were positioned inside recombinant block III (see Figure 3, bottom).

We also reanalyzed the recombination in *EBNA3A* using different approaches via nucleotide substitution methods and phylogenetic methods to confirm these findings. Twelve possible recombination events were identified, four of which were defined as misidentifications (Supplementary Tables S2 and S3). Of the eight remaining events, only one had statistical support (*p*-value ≤ 0.05) for all of the evaluation methods. This statistically supported event has the potential recombinant sequences NPC021 and NPC049, the major parent of the EBV-1 sequence RK_LCL_L4 from Papua New Guinea, and the minor parent of the EBV-2 sequence NPC049/NPC021 from China. (Supplementary Tables S5 and S4, event 1). This result is consistent, and reinforces the different patterns presented by the NPC021 and NPC049 sequences compared to EBV type 2.

## 4. Discussion

EBV nuclear antigens (EBNAs) are nuclear proteins expressed during latent infection. The six different latent antigens (EBNA1, 2, 3A, 3B, 3C, and LP) influence both the virus’ transcription and its host cell [37]. Several studies, using different methodological techniques, suggest the essential role of EBNA3A in B cell transformation by EBV [13,38,39,40,41,42,43,44]. Additionally, a recent study showed that mice infected with EBV deficient in EBNA3A and EBNA3C oncogenes had lower viral persistence levels than those infected with the wild-type virus [45].

EBV has a large genome with several repetitive regions posing a tremendous challenge to the assembly process. We found some EBV-2 sequences with unusual InDel patterns that could be representative of natural sequence variability, otherwise originating from sequence or assembly errors. Nevertheless, collecting genomic data is becoming a common issue in EBV studies. Many tumor-associated EBV genomes from different geographic areas have been sequenced, allowing access to the virus’ genetic information. Further studies that include a greater representation of little-explored geographic locations and the use of long-read sequencing technologies are necessary and imperative in order to improve and increase the confidence of the information that can be extracted from genomic analyses associated with EBV.

To dissect *EBNA3A* and characterize the gene variations, we recovered 994 *EBNA3A* sequences from EBV genomes. The scarcity of EBV sequences from South America urged us to recover and characterize the first 12 *EBNA3A* sequences of EBV-associated gastric carcinomas (EBV–GC) sequenced from Chile, generating more information about the South American sequences of these viruses of both EBV subtypes. No unique signatures were found among South American sequences. It will be necessary to recover more genome sequences from South American EBV in order to study the viral origin and evolution.

The EBNA3 family comprises EBNA3A, EBNA3B, and EBNA3C—non-redundant genes arranged in tandem and transcribed by the Cp or Wp promoters, originating along mRNA that is alternatively spliced [13,38]. These genes share a typical exon structure (a tandem array of exons: short 5′ exon and longer 3′ exon separated by a short intron) and a homology domain (~300 aa) N-terminal region [14]. The *EBNA3* genes possibly originated from duplication events of a single *EBNA3* ancestral gene [46]. Furthermore, EBNA3′s intron has stop codons in all reading frames and suboptimal splicing sites [47]. EBNA3′s intronic region, in contrast to its exons (30%), has a high sequence homology (95–98%) between EBV-1 and EBV-2 at the nucleotide level, indicating selective pressure [38,47]. We investigated *EBNA3A*’s intron nucleotide sequence for EBV-2-unique signatures. A five-nucleotide signature is a genetic marker of EBV-2 strains present in the intronic region, as is a deletion of three nucleotides. Therefore, this intron’s retention and its characteristic type signatures would be related to the fine-tuning expression of EBNA3′s genes [47]. *EBNA3A* phylogenetic reconstruction based on a short 5′ exon and longer 3′ exon of 1006 *EBNA3A* sequences could discriminate EBV types. In addition, EBV-2 is a more geographically restricted virus compared with EBV-1, reinforcing previous studies [48,49,50,51], and this is the first evidence-based sequence analysis of the presence of EBV-2 in Chile.

The presence of several nucleotide changes between EBV-1 and EBV-2 within the *EBNA3A* gene allows for a clear phylogenetic type definition. The EBNA3 genes are frequently a target of the immune system, which is a possible explanation for the notable presence of mutations in these genes [6,50,52]. Some deletions previously described exclusively in EBV-2 prototype AG876 [7] were confirmed in most EBV-2 strains.

The EBNA3 family contains several NLSs to guarantee its subcellular localization in the nucleus [20]. The NLSs of EBNA3 interact with cargo transporter proteins, such as importin alpha/beta-5, and regulate the protein’s transport into the nucleus through the nuclear pore complex (NPC) [53]. Regarding the potential NLSs identified in EBNA3: EBNA3A [21] has six potential NLSs; EBNA3B [19] has four potential NLSs; and EBNA3C [54] has five potential NLSs. Therefore, EBNA3A has the largest number of NLSs compared with the other EBNA3 genes.

Computational prediction analyses combined with site-direct mutagenesis experiments using GFP-tagged partial constructs showed that mutation in NLS3 or NLS4 alone was insufficient to exclude the EBNA3A protein from the nucleus. In contrast, mutations in both NLS3 and NLS4 resulted in the EBNA3A partial protein being restricted to the cytoplasm subcellular localization [21]. We report four nucleotide substitutions in both NLS3 and NLS4, present exclusively in the EBNA3A of EBV-2 strains. The NLS3 is a monopartite NLS pattern 7, which begins with proline, and contains three or four basic residues [19]. All EBV-2 EBNA3A sequences had the NLS3 TKHRRPP motif amino acid substitutions, resulting in a motif with a lack of proline. Likewise, all EBV-2 EBNA3A sequences had the NLS4 RAGKG motif amino acid substitution, resulting in a motif with fewer basic residues (only two basic residues). There are some pieces of evidence that proline substitution in NLS affects its function. (1) Mutagenesis analyses of the transcription factor c-Myc reveal which proline amino acid is crucial to efficiently importing the protein into the nucleus [55]. (2) Another study shows alteration in protein kinase CK2 interaction with importin by altering NLSs’ proline via mutagenesis [56]. Somehow, this could be affecting the EBNA3A’s traffic to the nucleus.

Like previous studies, we have identified recombinant regions in the *EBNA3A* gene [22,33,57,58,59,60]. We have delineated the effect of recombination in the *EBNA3A* gene, demonstrating that only EBV-2 lineages had recombination events predicted in ancestral nodes. The NLS3 and NLS4 amino acid changes are present inside recombination block III. These common ancestor strains could have undergone a recombination event that changed the NLS3 and NLS4 from canonical to non-canonical motifs.

As previously discussed in Coleman et al. [11], why EBV-2 is less efficient than EBV-1 in transforming B lymphocytes in vitro is intriguing. EBV-2 was detected infecting the T-cells of healthy Kenyan children [61], and displayed tropism to T cells in vitro [11]. In addition, Burgess et al. [19] hypothesized that the presence of multiple NLSs could confer different functions or specificities to different cell types in the EBNA3 family proteins.

Trying to solve one piece of this puzzle, we investigated the NLSs of EBNA3A. EBV-2 sequences simultaneously presented two non-canonical NLS motifs (NLS3 and NLS4), contrasting with the EBV-1 sequences with the six canonical NLS motifs. This finding evidences the presence of genetic patterns in NLS motifs associated with EBV-1 and EBV-2. Considering that EBNA3A plays an important role in EBV transformation, our findings suggest that the NLSs from EBNA3A could be contributing to enabling EBV-1 and EBV-2 to have distinct specificity to different cell types, but further experiments using recombinant viruses are necessary in order to confirm the impact of EBNA3A NLS mutations on cell tropism.

## 5. Conclusions

In summary, our study analyzed more than one thousand EBNA3A sequences from EBV-1 and EBV-2, including the first sequences of EBV-positive gastric cancer from Chile. We identified the presence of EBV-1 and EBV-2 circulating in Chile. At a global level, we determined signatures exclusive to the EBV-2 lineage within the EBNA3A gene in both exon and intron regions, and these lineage-specific signatures could serve as EBV type markers. Therefore, EBNA3A’s NLS genetic patterns associated with EBV types provide insights into their roles in different cell-type specificities, and provide new information about the traits of this protein in the EBV’s biology. These findings provide the motivation for experimental validation to confirm the impact of EBNA3A NLS mutations on cell-type specificities.

## Data Availability

The data presented in this study are openly available on GenBank at accession numbers [MW219146–MW219157].

## Supplementary Materials

The following are available online at https://www.mdpi.com/article/10.3390/cancers13112569/s1: Supplementary Table S1: List of sequences analyzed in this study, including metadata; Supplementary Table S2: List of sequences of each putative recombinant event identified by RDP4 and breakpoint prediction; Supplementary Table S3: RDP4 methods with supporting *p*-values for putative recombinant events in the EBNA3A gene; Supplementary Table S4: Characteristics of 24 South American EBV sequences; Supplementary Table S5: EBNA3A canonical NLS amino acid (aa) positions.; Supplementary Table S6: EBNA3A amino acid (aa) deletion patterns; and Supplementary Table S7: Putative recombination regions identified by Gubbins.
