# Supplementary material for: Genetic Patterns Found in the Nuclear Localization Signals (NLSs) Associated with EBV-1 and EBV-2 Provide New Insights into Their Contribution to Different Cell-Type Specificities
Source: Cancers (Basel). 2021 May 24;13(11):2569. doi: 10.3390/cancers13112569 (PMC8197229; doi:10.3390/cancers13112569)
Supplement: Supplementary file 1 [file cancers-13-02569-s001.zip › cancers-1234143-supplementary.pdf]

Genetic Patterns Found in the Nuclear Localization Signals (NLSs) Associated with EBV-1 and EBV-2 Provide New Insights into Their Contribution to Different Cell-Type Specificities

Louise Zanella [1,†], María Elena Reyes [1,2,†], Ismael Riquelme [3], Michel Abanto [4], Daniela León [1], Tamara Viscarra [1], Carmen Ili [1] and Priscilla Brebi [1,*]

Supplementary Table S1. List of sequences analyzed in this study, including metadata.

Nº	Accession nº	Isolate	Cancer/Normal	Country	Year
**1**	MG021312	AG876-GC1	GC	South Korea	2017
**2**	MH101970	NKTCL-SC14	NKTCL	China	2016
**3**	MH101960	NKTCL-SC04	NKTCL	China	2016
**4**	MH101959	NKTCL-SC03	NKTCL	China	2016
**5**	MH101961	NKTCL-SC05	NKTCL	China	2016
**6**	MH101966	NKTCL-SC10	NKTCL	China	2016
**7**	MH101971	NKTCL-SC15	NKTCL	China	2016
**8**	MH101965	NKTCL-SC09	NKTCL	China	2016
**9**	MH101964	NKTCL-SC08	NKTCL	China	2016
**10**	MH101963	NKTCL-SC07	NKTCL	China	2016
**11**	MH101962	NKTCL-SC06	NKTCL	China	2016
**12**	MH101958	NKTCL-SC02	NKTCL	China	2016
**13**	MH101957	NKTCL-SC01	NKTCL	China	2016
**14**	MK973062	rMSHJ	ND	Germany	2015
**15**	MK540371	NPCT013	NPC	China	2015
**16**	LS992258	P1-T1	PTLD-Tumor	United Kingdom	2015
**17**	MG298928	JC_VID41_BWA	CAEBV	USA	2015
**18**	MG298927	JC_VID14_BWA	CAEBV	USA	2015
**19**	MG298925	JC_27_BWA	CAEBV	USA	2015
**20**	MG298924	IMS_Saliva_90_BWA	Heath-Saliva	United Kingdom	2015
**21**	MG298922	IMS_Saliva_5_BWA	Heath-Saliva	United Kingdom	2015
**22**	MG298921	IMS_Saliva_216_BWA	Heath-Saliva	United Kingdom	2015
**23**	MG298920	IMS_Saliva_187_BWA	Heath-Saliva	United Kingdom	2015
**24**	MG298919	IMS_Saliva_155_BWA	Heath-Saliva	United Kingdom	2015
**25**	MG298826	AH_Saliva_9077	HS	Taiwan	2015
**26**	MG298825	AH_Saliva_8489	HS	Taiwan	2015
**27**	MG298824	AH_Saliva_8471	HS	Taiwan	2015
**28**	MG298823	AH_Saliva_8192	HS	Taiwan	2015
**29**	MG298877	JM_NKTLY_96.1	NKTCL	Indonesia	2015
**30**	MG298878	JM_NKTLY_98.1	NKTCL	Indonesia	2015
**31**	MH101969	NKTCL-SC13	NKTCL	China	2015
**32**	MH101968	NKTCL-SC12	NKTCL	China	2015

**33**	MH101967	NKTCL-SC11	NKTCL	China	2015
**34**	MG298900	JM_Saliva_20	BL	Uganda	2015
**35**	MG298898	JM_NPC_bru_L7	NPC	Indonesia	2015
**36**	MG298827	AH_Saliva_9316	HS	Taiwan	2015
**37**	MG298902	JM_Saliva_5	BL	Uganda	2015
**38**	MG298901	JM_Saliva_33	BL	Uganda	2015
**39**	MG298899	JM_Saliva_18	BL	Uganda	2015
**40**	MG298897	JM_NPC_bru_L5	NPC	Indonesia	2015
**41**	MG298896	JM_NPC_bru_L47	NPC	Indonesia	2015
**42**	MG298895	JM_NPC_bru_L42	NPC	Indonesia	2015
**43**	MG298894	JM_NPC_bru_L41	NPC	Indonesia	2015
**44**	MG298891	JM_NPC_bru_L3	NPC	Indonesia	2015
**45**	MG298890	JM_NPC_bru_L29	NPC	Indonesia	2015
**46**	MG298889	JM_NPC_bru_L2	NPC	Indonesia	2015
**47**	MG298888	JM_NPC_bru_L16	NPC	Indonesia	2015
**48**	MG298887	JM_NPC_bru_51	NPC	Indonesia	2015
**49**	MG298886	JM_NPC_bru_38	NPC	Indonesia	2015
**50**	MG298885	JM_NPC_bru_377	NPC	Indonesia	2015
**51**	MG298884	JM_NPC_bru_238	NPC	Indonesia	2015
**52**	MG298883	JM_NPC_bru_178	NPC	Indonesia	2015
**53**	MG298882	JM_NPC_biop_51	NPC	Indonesia	2015
**54**	MG298881	JM_NPC_biop_27	NPC	Indonesia	2015
**55**	MG298880	JM_NPC_biop_238	NPC	Indonesia	2015
**56**	MG298879	JM_NPC_biop_162	NPC	Indonesia	2015
**57**	MG298876	JM_NKTLY_218.1	NKTCL	Indonesia	2015
**58**	MG298849	IMS_Saliva_177	HS	United Kingdom	2015
**59**	MG298848	IMS_Saliva_170	HS	United Kingdom	2015
**60**	MG298847	IMS_Saliva_162	HS	United Kingdom	2015
**61**	MG298844	IMS_Saliva_10	HS	United Kingdom	2015
**62**	MG298871	JC_V006	CAEBV	USA	2015
**63**	MG298870	JC_V005	CAEBV	USA	2015
**64**	MG298869	JC_40	CAEBV	USA	2015
**65**	MG298868	JC_37	CAEBV	USA	2015
**66**	MG298867	JC_30_29	CAEBV	USA	2015
**67**	MG298866	JC_30_18	CAEBV	USA	2015
**68**	MG298863	IMS_Saliva_9	HS	United Kingdom	2015
**69**	MG298862	IMS_Saliva_71	HS	United Kingdom	2015
**70**	MG298861	IMS_Saliva_70	HS	United Kingdom	2015
**71**	MG298860	IMS_Saliva_6	HS	United Kingdom	2015
**72**	MG298859	IMS_Saliva_52	HS	United Kingdom	2015
**73**	MG298857	IMS_Saliva_31	HS	United Kingdom	2015
**74**	MG298856	IMS_Saliva_250	HS	United Kingdom	2015
**75**	MG298855	IMS_Saliva_248	HS	United Kingdom	2015
**76**	MG298854	IMS_Saliva_243	HS	United Kingdom	2015
**77**	MG298853	IMS_Saliva_231	HS	United Kingdom	2015
**78**	MG298850	IMS_Saliva_193	HS	United Kingdom	2015
**79**	MG298875	JM_LCL_SU	sLCL	Indonesia	2015
**80**	MG298874	JM_LCL_MU	sLCL	Indonesia	2015
**81**	MG298873	JM_LCL_IN	sLCL	Indonesia	2015

**82**	MG298872	JM_LCL_IK	sLCL	Indonesia	2015
**83**	MG298841	GK_LY65	BL-cell-line	Uganda	2015
**84**	MG298838	GK_BL72	BL-cell-line	North Africa	2015
**85**	MG298837	GK_BL67	BL-cell-line	France	2015
**86**	MG298836	GK_BL60	BL-cell-line	North Africa	2015
**87**	MG298835	GK_BL44	BL-cell-line	North Africa	2015
**88**	MG298834	GK_BL42	BL-cell-line	North Africa	2015
**89**	MG298833	GK_BL36	BL-cell-line	North Africa	2015
**90**	MG298832	GK_BL18	BL-cell-line	North Africa	2015
**91**	MG298830	GK_Akuba	BL-cell-line	Kenya	2015
**92**	MG298829	DF_Tonsil_T49	BL	Argentina	2015
**93**	MG298828	DF_Tonsil_T47	Blymphoma	Argentina	2015
**94**	MG298839	GK_Farage	BL-cell-line	USA	2015
**95**	MG298926	JC_39_BWA	CAEBV	USA	2015
**96**	MG298892	JM_NPC_bru_L37	NPC	Indonesia	2015
**97**	MG298865	JC_23	CAEBV	USA	2015
**98**	MG298858	IMS_Saliva_49	HS	United Kingdom	2015
**99**	MG298851	IMS_Saliva_204	HS	United Kingdom	2015
**100**	MG298846	IMS_Saliva_120	HS	United Kingdom	2015
**101**	MG298845	IMS_Saliva_12	HS	United Kingdom	2015
**102**	MG298843	GK_RUDU	BL-cell-line	Africa	2015
**103**	MG298840	GK_LY47	BL-cell-line	Uganda	2015
**104**	MG298917	DF_Tonsil_T156_BWA	sLCL	Argentina	2015
**105**	MG298916	RK_LCL_L5	sLCL	Papua New Guinea	2015
**106**	MG298913	RK_LCL_L24	sLCL	Papua New Guinea	2015
**107**	MG298912	RK_LCL_L2	sLCL	Papua New Guinea	2015
**108**	MG298910	RK_LCL_L12	sLCL	Papua New Guinea	2015
**109**	MG298909	RK_LCL_H35	sLCL	Papua New Guinea	2015
**110**	MG298908	RK_LCL_H16	sLCL	Papua New Guinea	2015
**111**	MG298907	RK_LCL_H12	sLCL	Papua New Guinea	2015
**112**	MG298911	RK_LCL_L19	sLCL	Papua New Guinea	2015
**113**	MG298923	IMS_Saliva_81_BWA	Heath-Saliva	United Kingdom	2015
**114**	MG298918	GK_LY91_BWA	BL-cell-line	Africa	2015
**115**	MG298915	RK_LCL_L4	sLCL	Papua New Guinea	2015
**116**	MG298914	RK_LCL_L3	sLCL	Papua New Guinea	2015
**117**	MG298906	M81_gDNA	cell-line	China	2015
**118**	MG298842	GK_PUT	BL-cell-line	Africa	2015
**119**	MG298831	GK_BL16	BL-cell-line	North Africa	2015
**120**	MG298905	JWBL43B	BL-cell-line	Africa	2015
**121**	MG298904	JWBL17A	BL-cell-line	Africa	2015
**122**	MG298903	JWBL121B	BL-cell-line	Africa	2015
**123**	MK973061	IM-3	IM-Blood-cell-line	Germany	2014
**124**	MK540432	NPCT074S	NPC	China	2014
**125**	MK540431	NPCT074	NPC	China	2014
**126**	MK540292	HS041	HS	China	2014
**127**	MK540291	HS039	HS	China	2014
**128**	MK540290	HS038	HS	China	2014
**129**	MK540289	HS037	HS	China	2014
**130**	MK540288	HS036	HS	China	2014

**131**	MK540287	HS035	HS	China	2014
**132**	LS992255	EBV30	HSOT	United Kingdom	2014
**133**	LS992252	EBV27	HSOT	United Kingdom	2014
**134**	LS992251	EBV25	HSOT	United Kingdom	2014
**135**	LS992250	EBV22	HSOT	United Kingdom	2014
**136**	LS992249	EBV19	HSOT	United Kingdom	2014
**137**	LS992248	EBV21	HSOT	United Kingdom	2014
**138**	MG021315	EBVaGC8-3	GC	China	2014
**139**	MG021314	EBVaGC8-2	GC	China	2014
**140**	MF547487	E1578_OWv7	IM	USA	2014
**141**	MF547486	E1578_BCv7	IM	USA	2014
**142**	MF547476	E1590_OWv7	IM	USA	2014
**143**	MF547475	E1590_BCv7	IM	USA	2014
**144**	MF547474	E1590_OWv1	IM	USA	2014
**145**	MF547473	E1590_BCv1	IM	USA	2014
**146**	MF547471	E1563_OWv7	IM	USA	2014
**147**	MF547460	E1587_OWv7	IM	USA	2014
**148**	MF547459	E1587_BCv7	IM	USA	2014
**149**	MF547456	E1583_OWv7	IM	USA	2014
**150**	MF547455	E1583_BCv7	IM	USA	2014
**151**	KT823509	LC4	LC	China	2014
**152**	KT823508	LC3	LC	China	2014
**153**	KT823506	LC1	LC	China	2014
**154**	KT273944	EBVaGC4	GC	China	2014
**155**	KT254013	EBVaGC3	GC	China	2014
**156**	MF547472	E1563_BCV7	IM	USA	2014
**157**	MG021305	YCCEL1-GC1	GC	China	2014
**158**	KT823507	LC2	LC	China	2014
**159**	MK540470	NPCT115	NPC	China	2013
**160**	MK540469	NPCT114	NPC	China	2013
**161**	MK540468	NPCT113	NPC	China	2013
**162**	MK540467	NPCT112	NPC	China	2013
**163**	MK540466	NPCT111	NPC	China	2013
**164**	MK540465	NPCT110	NPC	China	2013
**165**	MK540464	NPCT109	NPC	China	2013
**166**	MK540463	NPCT108	NPC	China	2013
**167**	MK540462	NPCT107	NPC	China	2013
**168**	MK540461	NPCT106	NPC	China	2013
**169**	MK540460	NPCT105	NPC	China	2013
**170**	MK540459	NPCT104	NPC	China	2013
**171**	MK540458	NPCT103	NPC	China	2013
**172**	MK540457	NPCT102	NPC	China	2013
**173**	MK540456	NPCT101	NPC	China	2013
**174**	MK540455	NPCT100	NPC	China	2013
**175**	MK540454	NPCT099	NPC	China	2013
**176**	MK540453	NPCT098	NPC	China	2013
**177**	MK540452	NPCT096	NPC	China	2013
**178**	MK540451	NPCT094	NPC	China	2013
**179**	MK540450	NPCT093	NPC	China	2013

**180**	MK540449	NPCT092	NPC	China	2013
**181**	MK540448	NPCT091	NPC	China	2013
**182**	MK540447	NPCT090	NPC	China	2013
**183**	MK540446	NPCT089	NPC	China	2013
**184**	MK540445	NPCT088	NPC	China	2013
**185**	MK540444	NPCT087	NPC	China	2013
**186**	MK540443	NPCT086	NPC	China	2013
**187**	MK540442	NPCT085	NPC	China	2013
**188**	MK540441	NPCT084	NPC	China	2013
**189**	MK540440	NPCT083	NPC	China	2013
**190**	MK540439	NPCT082	NPC	China	2013
**191**	MK540438	NPCT081	NPC	China	2013
**192**	MK540437	NPCT080	NPC	China	2013
**193**	MK540436	NPCT078	NPC	China	2013
**194**	MK540435	NPCT077	NPC	China	2013
**195**	MK540434	NPCT076	NPC	China	2013
**196**	MK540433	NPCT075	NPC	China	2013
**197**	MK540430	NPCT073	NPC	China	2013
**198**	MK540429	NPCT072	NPC	China	2013
**199**	MK540428	NPCT071	NPC	China	2013
**200**	MK540427	NPCT070	NPC	China	2013
**201**	MK540426	NPCT069	NPC	China	2013
**202**	MK540425	NPCT068	NPC	China	2013
**203**	MK540424	NPCT067	NPC	China	2013
**204**	MK540423	NPCT066	NPC	China	2013
**205**	MK540422	NPCT065	NPC	China	2013
**206**	MK540421	NPCT064	NPC	China	2013
**207**	MK540420	NPCT063	NPC	China	2013
**208**	MK540419	NPCT062	NPC	China	2013
**209**	MK540418	NPCT061	NPC	China	2013
**210**	MK540417	NPCT060	NPC	China	2013
**211**	MK540416	NPCT059	NPC	China	2013
**212**	MK540415	NPCT058M	NPC	China	2013
**213**	MK540414	NPCT058	NPC	China	2013
**214**	MK540413	NPCT057M	NPC	China	2013
**215**	MK540412	NPCT057	NPC	China	2013
**216**	MK540411	NPCT056M	NPC	China	2013
**217**	MK540410	NPCT056	NPC	China	2013
**218**	MK540409	NPCT055M	NPC	China	2013
**219**	MK540408	NPCT055	NPC	China	2013
**220**	MK540407	NPCT054M	NPC	China	2013
**221**	MK540406	NPCT054	NPC	China	2013
**222**	MK540405	NPCT053	NPC	China	2013
**223**	MK540404	NPCT052	NPC	China	2013
**224**	MK540403	NPCT050	NPC	China	2013
**225**	MK540401	NPCT048	NPC	China	2013
**226**	MK540400	NPCT047	NPC	China	2013
**227**	MK540399	NPCT046	NPC	China	2013
**228**	MK540398	NPCT045	NPC	China	2013

**229**	MK540397	NPCT043	NPC	China	2013
**230**	MK540396	NPCT042	NPC	China	2013
**231**	MK540395	NPCT041	NPC	China	2013
**232**	MK540394	NPCT040	NPC	China	2013
**233**	MK540393	NPCT039	NPC	China	2013
**234**	MK540392	NPCT038	NPC	China	2013
**235**	MK540391	NPCT037	NPC	China	2013
**236**	MK540390	NPCT036	NPC	China	2013
**237**	MK540389	NPCT035	NPC	China	2013
**238**	MK540388	NPCT033	NPC	China	2013
**239**	MK540387	NPCT032	NPC	China	2013
**240**	MK540386	NPCT031	NPC	China	2013
**241**	MK540385	NPCT029	NPC	China	2013
**242**	MK540384	NPCT028-2	NPC	China	2013
**243**	MK540383	NPCT027	NPC	China	2013
**244**	MK540382	NPCT025	NPC	China	2013
**245**	MK540381	NPCT024	NPC	China	2013
**246**	MK540380	NPCT023	NPC	China	2013
**247**	MK540379	NPCT022	NPC	China	2013
**248**	MK540377	NPCT020-2	NPC	China	2013
**249**	MK540376	NPCT019	NPC	China	2013
**250**	MK540375	NPCT018	NPC	China	2013
**251**	MK540374	NPCT017	NPC	China	2013
**252**	MK540373	NPCT015	NPC	China	2013
**253**	MK540372	NPCT014	NPC	China	2013
**254**	MK540370	NPCT012	NPC	China	2013
**255**	MK540369	NPCT011	NPC	China	2013
**256**	MK540368	NPCT010	NPC	China	2013
**257**	MK540367	NPCT009	NPC	China	2013
**258**	MK540366	NPCT008	NPC	China	2013
**259**	MK540365	NPCT007	NPC	China	2013
**260**	MK540364	NPCT006	NPC	China	2013
**261**	MK540363	NPCT005	NPC	China	2013
**262**	MK540361	NPCT003	NPC	China	2013
**263**	MK540360	NPCT002	NPC	China	2013
**264**	MK540359	NPCT001	NPC	China	2013
**265**	MK540358	NPCS054	NPC	China	2013
**266**	MK540357	NPCS052	NPC	China	2013
**267**	MK540356	NPCS051	NPC	China	2013
**268**	MK540354	NPCS049	NPC	China	2013
**269**	MK540353	NPCS048	NPC	China	2013
**270**	MG021310	Mutu-GC3	GC	Poland	2013
**271**	MG021307	AG876 -GC1	GC	South Korea	2013
**272**	MK540352	NPCS047	NPC	China	2013
**273**	MK540351	NPCS046	NPC	China	2013
**274**	MK540350	NPCS045	NPC	China	2013
**275**	MK540313	NPCP001	NPC	China	2013
**276**	MK540312	NNPCT005	NPC	China	2013
**277**	MK540311	NNPCT004	NPC	China	2013

**278**	MK540310	NNPCT003	NPC	China	2013
**279**	MK540309	NNPCT002	NPC	China	2013
**280**	MK540308	NNPCT001	NPC	China	2013
**281**	MK540307	NKLT007	NKTCL	China	2013
**282**	MK540306	NKLT006	NKTCL	China	2013
**283**	MK540305	NKLT004	NKTCL	China	2013
**284**	MK540304	NKLT003-2	NKTCL	China	2013
**285**	MK540302	NHS004	HS	China	2013
**286**	MK540301	NHS002	HS	China	2013
**287**	MK540300	HS057	HS	China	2013
**288**	MK540299	HS054	HS	China	2013
**289**	MK540298	HS053	HS	China	2013
**290**	MK540297	HS052	HS	China	2013
**291**	MK540296	HS051	HS	China	2013
**292**	MK540295	HS050	HS	China	2013
**293**	MK540294	HS048	HS	China	2013
**294**	MK540293	HS045	HS	China	2013
**295**	MK540286	HS034	HS	China	2013
**296**	MK540281	HS025	HS	China	2013
**297**	MK540265	HS003	HS	China	2013
**298**	MK540264	HS001	HS	China	2013
**299**	MK540263	HLT011	HL	China	2013
**300**	MK540262	HLT010	HL	China	2013
**301**	MK540256	GCT014	GC	China	2013
**302**	MK540255	GCT013	GC	China	2013
**303**	MK540254	GCT012	GC	China	2013
**304**	MK540253	GCT011	GC	China	2013
**305**	MK540252	GCT010	GC	China	2013
**306**	MK540251	GCT009	GC	China	2013
**307**	MK540250	GCT007	GC	China	2013
**308**	MK540249	GCT006	GC	China	2013
**309**	MK540248	GCT005	GC	China	2013
**310**	MK540247	GCT004	GC	China	2013
**311**	MK540246	GCT003	GC	China	2013
**312**	MK540245	GCT002	GC	China	2013
**313**	MK540244	GCT001	GC	China	2013
**314**	MK540242	BLT002	BL	China	2013
**315**	MK540241	BLT001	BL	China	2013
**316**	AP019187	UPN98_PBMC	CAEBV	Japan	2013
**317**	AP019181	UPN874_PBMC	NKTCL	Japan	2013
**318**	AP019180	UPN584_PBMC	CAEBV	Japan	2013
**319**	AP019179	UPN582_PBMC	CAEBV	Japan	2013
**320**	AP019178	UPN573_PBMC	CAEBV	Japan	2013
**321**	AP019172	UPN5575_PBMC	NKTCL	Japan	2013
**322**	AP019169	UPN520_PBMC	CAEBV	Japan	2013
**323**	AP019166	UPN5087_PBMC	DLBCL	Japan	2013
**324**	AP019165	UPN5_PBMC	IM	Japan	2013
**325**	AP019164	UPN498_PBMC	CAEBV	Japan	2013
**326**	AP019162	UPN495_PBMC	CAEBV	Japan	2013

**327**	AP019158	UPN484_PBMC	CAEBV	Japan	2013
**328**	AP019156	UPN463_PBMC	CAEBV	Japan	2013
**329**	AP019151	UPN417_PBMC	CAEBV	Japan	2013
**330**	AP019150	UPN417_CD56+	CAEBV	Japan	2013
**331**	AP019149	UPN417_CD3+	CAEBV	Japan	2013
**332**	AP019148	UPN4137_tissue	LM	Japan	2013
**333**	AP019142	UPN404_PBMC	CAEBV	Japan	2013
**334**	AP019141	UPN404_CD56+	CAEBV	Japan	2013
**335**	AP019140	UPN404_CD3+	CAEBV	Japan	2013
**336**	AP019136	UPN3707_PBMC	DLBCL	Japan	2013
**337**	AP019135	UPN366_CD56+	CAEBV	Japan	2013
**338**	AP019134	UPN366_CD3+	CAEBV	Japan	2013
**339**	AP019125	UPN3198_PBMC	NKTCL	Japan	2013
**340**	AP019124	UPN311_CD56+	CAEBV	Japan	2013
**341**	AP019123	UPN310_CD56+	CAEBV	Japan	2013
**342**	AP019118	UPN301_CD56+	CAEBV	Japan	2013
**343**	AP019115	UPN27_PBMC	PTLD	Japan	2013
**344**	AP019114	UPN267_PBMC	CAEBV	Japan	2013
**345**	AP019111	UPN265_PBMC	ANCL	Japan	2013
**346**	AP019110	UPN264_PBMC	CAEBV	Japan	2013
**347**	AP019108	UPN262_PBMC	CAEBV	Japan	2013
**348**	AP019105	UPN26_PBMC	PTLD	Japan	2013
**349**	AP019104	UPN259_PBMC	CAEBV	Japan	2013
**350**	AP019102	UPN257_PBMC	CAEBV	Japan	2013
**351**	AP019101	UPN256_PBMC	CAEBV	Japan	2013
**352**	AP019096	UPN251_PBMC	CAEBV	Japan	2013
**353**	AP019087	UPN1926_PBMC	NKTCL	Japan	2013
**354**	AP019083	UPN1833_tissue	LE	Japan	2013
**355**	AP019080	UPN18_PBMC	PTLD	Japan	2013
**356**	AP019076	UPN1756_PBMC	NKTCL	Japan	2013
**357**	AP019070	UPN140_tumor	CAEBV	Japan	2013
**358**	AP019067	UPN138_tumor	CAEBV	Japan	2013
**359**	AP019066	UPN138_PBMC	CAEBV	Japan	2013
**360**	AP019065	UPN136_tumor	CAEBV	Japan	2013
**361**	AP019064	UPN136_PBMC	CAEBV	Japan	2013
**362**	AP019063	UPN134_tumor	CAEBV	Japan	2013
**363**	AP019060	UPN132_PBMC	CAEBV	Japan	2013
**364**	AP019053	UPN123_tumor	CAEBV	Japan	2013
**365**	AP019051	UPN1201_PBMC	NKTCL	Japan	2013
**366**	AP019050	UPN12_PBMC	IM	Japan	2013
**367**	AP019049	UPN114_tumor	CAEBV	Japan	2013
**368**	AP019048	UPN114_PBMC	CAEBV	Japan	2013
**369**	AP019044	UPN112_PBMC	CAEBV	Japan	2013
**370**	AP019042	UPN1113_PBMC	NKTCL	Japan	2013
**371**	AP019040	UPN1110_PBMC	NKTCL	Japan	2013
**372**	AP019038	UPN1106_PBMC	NKTCL	Japan	2013
**373**	AP019036	UPN1102_PBMC	NKTCL	Japan	2013
**374**	AP019030	UPN107_PBMC	CAEBV	Japan	2013
**375**	AP019029	UPN106_PBMC	CAEBV	Japan	2013

**376**	AP019028	UPN105_tumor	CAEBV	Japan	2013
**377**	AP019027	UPN105_PBMC	CAEBV	Japan	2013
**378**	AP019024	UPN1008_PBMC	DLBCL	Japan	2013
**379**	AP019018	SNT8_cell	cell-line	Japan	2013
**380**	AP019014	SNK10_cell	cell-line	Japan	2013
**381**	AP019013	SNK1_cell	cell-line	Japan	2013
**382**	LS992245	EBV15	HSOT	United Kingdom	2013
**383**	MG021313	EBVaGC8-1	GC	South Korea	2013
**384**	MG021308	Mutu-GC1	GC	Poland	2013
**385**	MG021309	Mutu-GC2	GC	USA	2013
**386**	MF547490	E1577_OWv1	IM	USA	2013
**387**	MF547489	E1577_BCv1	IM	USA	2013
**388**	MF547485	E1578_BCv1	IM	USA	2013
**389**	MF547470	E1563_OWv1	IM	USA	2013
**390**	MF547469	E1548_OWv7	IM	USA	2013
**391**	MF547468	E1548_BCv7	IM	USA	2013
**392**	MF547467	E1548_OWv1	IM	USA	2013
**393**	MF547466	E1548_BCv1	IM	USA	2013
**394**	MF547465	E1536_BCv7	IM	USA	2013
**395**	MF547463	E1563_BCv1	IM	USA	2013
**396**	MF547464	E1536_OWv7	IM	USA	2013
**397**	MF547458	E1587_OWv1	IM	USA	2013
**398**	MF547457	E1587_BCv1	IM	USA	2013
**399**	MF547454	E1583_OWv1	IM	USA	2013
**400**	MF547453	E1583_BCv1	IM	USA	2013
**401**	LC150743	HNNPC8	NPC	China	2013
**402**	LC150741	HNNPC6	NPC	China	2013
**403**	LC150338	HNNPC5	NPC	China	2013
**404**	LC150327	HNNPC3	NPC	China	2013
**405**	LC149491	HNNPC2	NPC	China	2013
**406**	LC137018	HNNPC1	NPC	China	2013
**407**	KT273943	EBVaGC2	GC	China	2013
**408**	LC150337	HNNPC4	NPC	China	2013
**409**	MG021311	Mutu-GC4	GC	Ukraine	2013
**410**	AP019079	UPN1789_PBMC	NKTCL	Japan	2013
**411**	AP019061	UPN132_tumor	CAEBV	Japan	2013
**412**	AP019032	UPN108_tumor	CAEBV	Japan	2013
**413**	MG021317	EBVaGC5-1	GC	South Korea	2013
**414**	MG021316	HKNPC6-GC1	GC	Vietnam	2013
**415**	MG021306	YCCEL1-GC2	GC	Poland	2013
**416**	MF547488	E1578_OWv1	IM	USA	2013
**417**	LC150742	HNNPC7	NPC	China	2013
**418**	KT273942	EBVaGC1	GC	China	2013
**419**	AP019037	UPN1104_PBMC	NKTCL	Japan	2013
**420**	AP019077	UPN1757_PBMC	NKTCL	Japan	2013
**421**	AP019155	UPN4314_PBMC	DLBCL	Japan	2013
**422**	AP019188	UPN99_PBMC	CAEBV	Japan	2013
**423**	AP019186	UPN97_tumor	CAEBV	Japan	2013
**424**	AP019185	UPN97_PBMC	CAEBV	Japan	2013

**425**	AP019184	UPN93_gdT-2	CAEBV	Japan	2013
**426**	AP019177	UPN571_CD56+	CAEBV	Japan	2013
**427**	AP019176	UPN571_CD3+	CAEBV	Japan	2013
**428**	AP019175	UPN563_CD56+	CAEBV	Japan	2013
**429**	AP019174	UPN563_CD3+	CAEBV	Japan	2013
**430**	AP019173	UPN563_CD19+	CAEBV	Japan	2013
**431**	AP019171	UPN554_PBMC	CAEBV	Japan	2013
**432**	AP019170	UPN539_PBMC	CAEBV	Japan	2013
**433**	AP019168	UPN519_PBMC	CAEBV	Japan	2013
**434**	AP019163	UPN497_PBMC	CAEBV	Japan	2013
**435**	AP019161	UPN494_CD56+	CAEBV	Japan	2013
**436**	AP019160	UPN494_CD3+	CAEBV	Japan	2013
**437**	AP019159	UPN486_PBMC	CAEBV	Japan	2013
**438**	AP019157	UPN470_PBMC	CAEBV	Japan	2013
**439**	AP019154	UPN431_CD56+	CAEBV	Japan	2013
**440**	AP019153	UPN431_CD3+	CAEBV	Japan	2013
**441**	AP019152	UPN431_CD19+	CAEBV	Japan	2013
**442**	AP019147	UPN412_PBMC	CAEBV	Japan	2013
**443**	AP019145	UPN412_CD3+	CAEBV	Japan	2013
**444**	AP019144	UPN405_CD56+	CAEBV	Japan	2013
**445**	AP019139	UPN3755_tissue	LE	Japan	2013
**446**	AP019133	UPN365_CD56+	CAEBV	Japan	2013
**447**	AP019131	UPN3621_PBMC	NKTCL	Japan	2013
**448**	AP019130	UPN3448_PBMC	DLBCL	Japan	2013
**449**	AP019129	UPN3300_PBMC	NKTCL	Japan	2013
**450**	AP019128	UPN326_PBMC	CAEBV	Japan	2013
**451**	AP019126	UPN322_CD3+	CAEBV	Japan	2013
**452**	AP019122	UPN307_CD56+	CAEBV	Japan	2013
**453**	AP019121	UPN3065_PBMC	NKTCL	Japan	2013
**454**	AP019119	UPN303_CD56+	CAEBV	Japan	2013
**455**	AP019113	UPN266_PBMC	CAEBV	Japan	2013
**456**	AP019109	UPN263_PBMC	CAEBV	Japan	2013
**457**	AP019107	UPN261_PBMC	CAEBV	Japan	2013
**458**	AP019106	UPN260_PBMC	CAEBV	Japan	2013
**459**	AP019103	UPN258_PBMC	CAEBV	Japan	2013
**460**	AP019100	UPN255_PBMC	CAEBV	Japan	2013
**461**	AP019099	UPN254_PBMC	CAEBV	Japan	2013
**462**	AP019098	UPN253_PBMC	CAEBV	Japan	2013
**463**	AP019097	UPN252_PBMC	CAEBV	Japan	2013
**464**	AP019095	UPN25_PBMC	PTLD	Japan	2013
**465**	AP019094	UPN24_PBMC	PTLD	Japan	2013
**466**	AP019092	UPN23_PBMC	PTLD	Japan	2013
**467**	AP019091	UPN22_PBMC	PTLD	Japan	2013
**468**	AP019090	UPN21_PBMC	PTLD	Japan	2013
**469**	AP019089	UPN20_PBMC	PTLD	Japan	2013
**470**	AP019088	UPN2_PBMC	IM	Japan	2013
**471**	AP019086	UPN1901_tissue	LM	Japan	2013
**472**	AP019084	UPN185_PBMC	CAEBV	Japan	2013
**473**	AP019082	UPN1813_PBMC	NKTCL	Japan	2013

**474**	AP019081	UPN1802_PBMC	NKTCL	Japan	2013
**475**	AP019078	UPN1758_PBMC	NKTCL	Japan	2013
**476**	AP019074	UPN15_PBMC	PTLD	Japan	2013
**477**	AP019073	UPN142_PBMC	CAEBV	Japan	2013
**478**	AP019072	UPN142_gdT-2	CAEBV	Japan	2013
**479**	AP019071	UPN142_gdT-1	CAEBV	Japan	2013
**480**	AP019059	UPN130_tumor	CAEBV	Japan	2013
**481**	AP019058	UPN130_PBMC	CAEBV	Japan	2013
**482**	AP019057	UPN13_PBMC	IM	Japan	2013
**483**	AP019055	UPN128_PBMC	CAEBV	Japan	2013
**484**	AP019054	UPN125_PBMC	CAEBV	Japan	2013
**485**	AP019052	UPN1202_PBMC	NKTCL	Japan	2013
**486**	AP019047	UPN113_PBMC	CAEBV	Japan	2013
**487**	AP019043	UPN1119_PBMC	NKTCL	Japan	2013
**488**	AP019041	UPN1111_PBMC	NKTCL	Japan	2013
**489**	AP019039	UPN111_PBMC	CAEBV	Japan	2013
**490**	AP019035	UPN1101_PBMC	NKTCL	Japan	2013
**491**	AP019034	UPN110_PBMC	CAEBV	Japan	2013
**492**	AP019033	UPN109_PBMC	CAEBV	Japan	2013
**493**	AP019026	UPN104_PBMC	CAEBV	Japan	2013
**494**	AP019025	UPN102_PBMC	CAEBV	Japan	2013
**495**	AP019020	UPN1003_PBMC	DLBCL	Japan	2013
**496**	AP019019	UPN1002_PBMC	DLBCL	Japan	2013
**497**	AP019017	SNT16_cell	cell-line	Japan	2013
**498**	AP019016	SNT15_cell	cell-line	Japan	2013
**499**	AP019015	SNT13_cell	cell-line	Japan	2013
**500**	AP019012	KAI3_cell	cell-line	Japan	2013
**501**	AP019132	UPN365_CD3+	CAEBV	Japan	2013
**502**	AP019120	UPN306_CD56+	CAEBV	Japan	2013
**503**	AP019068	UPN14_PBMC	PTLD	Japan	2013
**504**	AP019146	UPN412_CD56+	CAEBV	Japan	2013
**505**	AP019137	UPN375_CD3+	CAEBV	Japan	2013
**506**	AP019085	UPN19_PBMC	PTLD	Japan	2013
**507**	AP019075	UPN17_PBMC	PTLD	Japan	2013
**508**	AP019138	UPN375_CD56+	CAEBV	Japan	2013
**509**	MK540402	NPCT049	NPC	China	2013
**510**	MK540378	NPCT021	NPC	China	2013
**511**	MK540362	NPCT004	NPC	China	2012
**512**	MK540355	NPCS050	NPC	China	2012
**513**	MK540349	NPCS044	NPC	China	2012
**514**	MK540348	NPCS042	NPC	China	2012
**515**	MK540347	NPCS040	NPC	China	2012
**516**	MK540346	NPCS039	NPC	China	2012
**517**	MK540345	NPCS038	NPC	China	2012
**518**	MK540344	NPCS035	NPC	China	2012
**519**	MK540343	NPCS034	NPC	China	2012
**520**	MK540342	NPCS033	NPC	China	2012
**521**	MK540341	NPCS031	NPC	China	2012
**522**	MK540340	NPCS030	NPC	China	2012

**523**	MK540339	NPCS029	NPC	China	2012
**524**	MK540338	NPCS028	NPC	China	2012
**525**	MK540337	NPCS027	NPC	China	2012
**526**	MK540336	NPCS026	NPC	China	2012
**527**	MK540303	NKLT002	NKTCL	China	2012
**528**	MK540285	HS033	HS	China	2012
**529**	MK540284	HS032	HS	China	2012
**530**	MK540280	HS024	HS	China	2012
**531**	MK540279	HS023	HS	China	2012
**532**	MK540278	HS021	HS	China	2012
**533**	MK540274	HS016	HS	China	2012
**534**	MK540273	HS015	HS	China	2012
**535**	MK540272	HS014	HS	China	2012
**536**	MK540271	HS013	HS	China	2012
**537**	MK540261	HLT007	HL	China	2012
**538**	MK540260	HLT006	HL	China	2012
**539**	MK540259	HLT005	HL	China	2012
**540**	KC440852	K4123-MiEBV	sLCL	USA	2012
**541**	LS992257	P2-T1	PTLD-Tumor	United Kingdom	2012
**542**	LS992244	EBV13	HSOT	United Kingdom	2012
**543**	LS992243	EBV9	HSOT	United Kingdom	2012
**544**	LS992242	EBV7	HSOT	United Kingdom	2012
**545**	LS992239	EBV6	HSOT	United Kingdom	2012
**546**	MF547462	E1536_OWv1	IM	USA	2012
**547**	MF547461	E1536_BCv1	IM	USA	2012
**548**	AB850654	HN15	NPC	China	2012
**549**	KX674065	GDGC2	GC-cell-line	China	2012
**550**	KX125051	GC-EBV2	GC	China	2012
**551**	KC440851	K4123-Mi	sLCL	USA	2012
**552**	MK540335	NPCS025	NPC	China	2011
**553**	MK540334	NPCS024	NPC	China	2011
**554**	MK540333	NPCS023	NPC	China	2011
**555**	MK540332	NPCS022	NPC	China	2011
**556**	MK540331	NPCS021	NPC	China	2011
**557**	MK540330	NPCS019	NPC	China	2011
**558**	MK540329	NPCS018	NPC	China	2011
**559**	MK540328	NPCS017	NPC	China	2011
**560**	MK540327	NPCS016	NPC	China	2011
**561**	MK540326	NPCS014	NPC	China	2011
**562**	MK540325	NPCS013	NPC	China	2011
**563**	MK540324	NPCS012	NPC	China	2011
**564**	MK540323	NPCS011	NPC	China	2011
**565**	MK540322	NPCS010	NPC	China	2011
**566**	MK540321	NPCS009	NPC	China	2011
**567**	MK540320	NPCS008	NPC	China	2011
**568**	MK540319	NPCS007	NPC	China	2011
**569**	MK540318	NPCS006	NPC	China	2011
**570**	MK540317	NPCS005	NPC	China	2011
**571**	MK540316	NPCS003-2	NPC	China	2011

**572**	MK540315	NPCS002	NPC	China	2011
**573**	MK540314	NPCS001	NPC	China	2011
**574**	MK540283	HS029	HS	China	2011
**575**	MK540282	HS027	HS	China	2011
**576**	MK540270	HS012	HS	China	2011
**577**	MK540269	HS011	HS	China	2011
**578**	MK540268	HS009	HS	China	2011
**579**	MK540267	HS008	HS	China	2011
**580**	MK540258	HLT002	HL	China	2011
**581**	MK540257	HLT001	HL	China	2011
**582**	LS992256	P3-T1	PTLD-Tumor	United Kingdom	2011
**583**	MF547484	E1503_OWv7	IM	USA	2011
**584**	MF547483	E1503_BCv7	IM	USA	2011
**585**	MF547480	E1492_OWv7	IM	USA	2011
**586**	MF547479	E1492_BCv7	IM	USA	2011
**587**	KX674064	GDGC1	GC-cell-line	China	2011
**588**	KX125050	GC-EBV1	GC	China	2011
**589**	MW219155	CHI66	GC	Chile	2011
**590**	MW219154	CHI62	GC	Chile	2011
**591**	MK540277	HS020	HS	China	2010
**592**	MK540276	HS019	HS	China	2010
**593**	MK540275	HS018	HS	China	2010
**594**	MK540266	HS007	HS	China	2010
**595**	MF547482	E1503_OWv1	IM	USA	2010
**596**	MF547481	E1503_BCv1	IM	USA	2010
**597**	MF547478	E1492_OWv1	IM	USA	2010
**598**	MF547477	E1492_BCv1	IM	USA	2010
**599**	KT273949	EBVaGC9	GC	China	2010
**600**	KT273948	EBVaGC8	GC	China	2010
**601**	KP735248	GC1	GC-cell-line	South Korea	2010
**602**	KX674067	YCCEL1	GC-cell-line	South Korea	2010
**603**	KX125053	YCCEL1	GC-cell-line	South Korea	2010
**604**	HQ020558	GD2	NPC-tumor	China	2009
**605**	KT273947	EBVaGC7	GC	China	2009
**606**	KT273946	EBVaGC6	GC	China	2009
**607**	KT273945	EBVaGC5	GC	China	2009
**608**	MW219146	CHI29	GC	Chile	2009
**609**	MW219148	CHI33	GC	Chile	2009
**610**	MW219149	CHI34	GC	Chile	2009
**611**	MW219151	CHI47	GC	Chile	2009
**612**	MW219152	CHI53	GC	Chile	2009
**613**	MW219153	CHI55	GC	Chile	2009
**614**	MW219147	CHI32	GC	Chile	2009
**615**	MW219150	CHI39	GC	Chile	2009
**616**	KF992571	HKNPC9	NPC-tumor	China	2008
**617**	KF992570	HKNPC8	NPC	China	2008
**618**	KF992567	HKNPC5	NPC	China	2008
**619**	KF992566	HKNPC4	NPC	China	2008
**620**	KF992565	HKNPC3	NPC	China	2008

**621**	KF992564	HKNPC2	NPC	China	2008
**622**	JQ009376	HKNPC1	NPC-tumor	China	2008
**623**	KX125052	SNU-719	GC-cell-line	South Korea	2008
**624**	KX674066	SNU-719	GC-cell-line	South Korea	2008
**625**	KF992569	HKNPC7	NPC	China	2008
**626**	KF992568	HKNPC6	NPC	China	2008
**627**	MW219156	CHI127	GC	Chile	2008
**628**	MW219157	CHI130	GC	Chile	2008
**629**	LS992240	P4-T1	PTLD-Tumor	United Kingdom	2003
**630**	MK540243	C666	NPC-cell-line	China	1998
**631**	KJ411974	C666-1	NPC-cell-line	China	1998
**632**	KC617875	C666-1	NPC-cell-line	China	1998
**633**	AB828190	1LGY-C666-1	NPC	China	1998
**634**	LN827525	C666-1	NPC-cell-line	China	1998
**635**	AY961628	GD1	NPC-saliva	China	1998
**636**	KP968263	H058015C	BL	Ghana	1990
**637**	NC_007605	B95-8.Raji	IM-cell-line	USA	1984
**638**	AJ507799	B95-8.Raji	IM-cell-line	USA	1984
**639**	V01555	B95-8	IM-cell-line	USA	1984
**640**	KR063342	H03753A	BL	Ghana	1981
**641**	KP968264	H002213	BL	Ghana	1981
**642**	KT001103	SG	BL	Argentina	1981
**643**	KP968261	HU11393	BL	Ghana	1980
**644**	KP968258	MP	BL	Brazil	1980
**645**	KP968257	CCH	BL	Brazil	1980
**646**	KR063345	FNR	BL	Brazil	1980
**647**	KR063344	RPF	BL	Brazil	1980
**648**	KR063343	CV-ARG	BL	Argentina	1980
**649**	KP968260	VGO	BL	Brazil	1980
**650**	KP968259	SCL	BL	Brazil	1980
**651**	KT001102	VA	BL	Argentina	1980
**652**	KP968262	H018436D	BL	Ghana	1976
**653**	KF373730	M81	NPC	China	1970
**654**	MG298864	JC_2_CAEBV	ND	ND	ND
**655**	MH837528	NOSB	ND	France	ND
**656**	MH837527	PTLB3	ND	France	ND
**657**	MH837526	PTBL2	PTBL	France	ND
**658**	MH837525	PTBL1	PTBL	France	ND
**659**	MH837524	NKTL2	NKTL	France	ND
**660**	MH837523	DLBCL4	DLBCL	France	ND
**661**	MH837522	DLBCL2	DLBCL	France	ND
**662**	MH837521	CTCL1	AIL	France	ND
**663**	MH837520	ARL2	ARL	France	ND
**664**	MH837519	AIL16	AIL	France	ND
**665**	MH837518	AIL15	AIL	France	ND
**666**	MH837517	AIL14	AIL	France	ND
**667**	MH837516	AIL13	AIL	France	ND
**668**	MH837515	AIL7	AIL	France	ND
**669**	MH837514	AIL5	AIL	France	ND

**670**	MH837513	AIL2	AIL	France	ND
**671**	MH837512	AIL1	AIL	France	ND
**672**	MH590573	HKNPC62	NPC	China	ND
**673**	MH144221	NKTCL-SG10	NKTCL	Singapore	ND
**674**	MH144219	NKTCL-SG08	NKTCL	Singapore	ND
**675**	MH144218	NKTCL-SG07	NKTCL	Singapore	ND
**676**	MH144217	NKTCL-SG06	NKTCL	Singapore	ND
**677**	MH144214	NKTCL-SG03	NKTCL	Singapore	ND
**678**	MH590579	SNU719	GC-cell-line	Korea	ND
**679**	MH590578	NPC43	NPC-cell-line	China	ND
**680**	MH590577	M81	NPC-cell-line	China	ND
**681**	MH590575	C6661	NPC-cell-line	China	ND
**682**	MH590571	HKNPC60	NPC	China	ND
**683**	MH590570	HKNPC59	NPC	China	ND
**684**	MH590569	HKNPC58	NPC	China	ND
**685**	MH590568	HKNPC57	NPC	China	ND
**686**	MH590566	HKNPC55	NPC	China	ND
**687**	MH590565	HKNPC54	NPC	China	ND
**688**	MH590564	HKNPC53	NPC	China	ND
**689**	MH590560	HKNPC49	NPC	China	ND
**690**	MH590559	HKNPC48	NPC	China	ND
**691**	MH590557	HKNPC46	NPC	China	ND
**692**	MH590556	HKNPC45	NPC	China	ND
**693**	MH590555	HKNPC44	NPC	China	ND
**694**	MH590554	HKNPC43	NPC	China	ND
**695**	MH590553	HKNPC42	NPC	China	ND
**696**	MH590552	HKNPC41	NPC	China	ND
**697**	MH590551	HKNPC40	NPC	China	ND
**698**	MH590548	HKNPC37	NPC	China	ND
**699**	MH590545	HKNPC34	NPC	China	ND
**700**	MH590543	HKNPC32	NPC	China	ND
**701**	MH590542	HKNPC31	NPC	China	ND
**702**	MH590541	HKNPC30	NPC	China	ND
**703**	MH590540	HKNPC29	NPC	China	ND
**704**	MH590538	HKNPC27	NPC	China	ND
**705**	MH590536	HKNPC25	NPC	China	ND
**706**	MH590535	HKNPC24	NPC	China	ND
**707**	MH590534	HKNPC23	NPC	China	ND
**708**	MH590532	HKNPC21	NPC	China	ND
**709**	MH590531	HKNPC20	NPC	China	ND
**710**	MH590527	HKNPC16	NPC	China	ND
**711**	MH590525	HKNPC14	NPC	China	ND
**712**	MH590524	HKNPC13	NPC	China	ND
**713**	MH590523	HKNPC12	NPC	China	ND
**714**	MH590522	HKNPC11	NPC	China	ND
**715**	MH590521	HKNPC10	NPC	China	ND
**716**	MH590520	HKNPC9	NPC	China	ND
**717**	MH590519	HKNPC8	NPC	China	ND
**718**	MH590516	HKNPC5	NPC	China	ND

**719**	MH590515	HKNPC4	NPC	China	ND
**720**	MH590514	HKNPC3	NPC	China	ND
**721**	MH590513	HKNPC2	NPC	China	ND
**722**	MH590512	HKNPC1	NPC	China	ND
**723**	MH590509	HKHD140	HS	China	ND
**724**	MH590507	HKHD138	HS	China	ND
**725**	MH590504	HKHD135	HS	China	ND
**726**	MH590502	HKHD133	HS	China	ND
**727**	MH590500	HKHD131	HS	China	ND
**728**	MH590499	HKHD130	HS	China	ND
**729**	MH590498	HKHD129	HS	China	ND
**730**	MH590497	HKHD128	HS	China	ND
**731**	MH590484	HKHD115	HS	China	ND
**732**	MH590482	HKHD113	HS	China	ND
**733**	MH590480	HKHD111	HS	China	ND
**734**	MH590479	HKHD110	HS	China	ND
**735**	MH590474	HKHD105	HS	China	ND
**736**	MH590471	HKHD102	HS	China	ND
**737**	MH590470	HKHD101	HS	China	ND
**738**	MH590469	HKHD100	HS	China	ND
**739**	MH590463	HKHD94	HS	China	ND
**740**	MH590457	HKHD88	HS	China	ND
**741**	MH590456	HKHD87	HS	China	ND
**742**	MH590454	HKHD85	HS	China	ND
**743**	MH590453	HKHD84	HS	China	ND
**744**	MH590447	HKHD78	HS	China	ND
**745**	MH590442	HKHD73	HS	China	ND
**746**	MH590433	HKHD64	HS	China	ND
**747**	MH590426	HKHD57	HS	China	ND
**748**	MH590423	HKHD54	HS	China	ND
**749**	MH590420	HKHD51	HS	China	ND
**750**	MH590418	HKHD49	HS	China	ND
**751**	MH590416	HKHD47	HS	China	ND
**752**	MH590415	HKHD46	HS	China	ND
**753**	MH590414	HKHD45	HS	China	ND
**754**	MH590409	HKHD40	HS	China	ND
**755**	MH590408	HKHD39	HS	China	ND
**756**	MH590407	HKHD38	HS	China	ND
**757**	MH590406	HKHD37	HS	China	ND
**758**	MH590403	HKHD34	HS	China	ND
**759**	MH590402	HKHD33	HS	China	ND
**760**	MH590399	HKHD30	HS	China	ND
**761**	MH590395	HKHD26	HS	China	ND
**762**	MH590389	HKHD20	HS	China	ND
**763**	MH590387	HKHD18	HS	China	ND
**764**	MH590386	HKHD17	HS	China	ND
**765**	MH590379	HKHD10	HS	China	ND
**766**	MH590377	HKHD8	HS	China	ND
**767**	MH590375	HKHD6	HS	China	ND

**768**	MH590372	HKHD3	HS	China	ND
**769**	MH590371	HKHD2	HS	China	ND
**770**	MH590370	HKHD1	HS	China	ND
**771**	MH883786	P12-1026	IM	Japan	ND
**772**	KC207814	Mutu	BL-cell-line	Kenya	ND
**773**	KC207813	Akata	BL-cell-line	Japan	ND
**774**	MH883781	P6-1751	IM	Japan	ND
**775**	MH883779	P4-T1	PTLD-Tumor	United Kingdom	ND
**776**	MH883777	P3-T1	PTLD-Tumor	United Kingdom	ND
**777**	MH883775	P2-T1	PTLD-Tumor	United Kingdom	ND
**778**	MH883773	P1-T1	PTLD-Tumor	United Kingdom	ND
**779**	KF717093	Raji	BL-cell-line	Nigeria	ND
**780**	MH590576	Jijoye	BL-cell-line	USA	ND
**781**	LN827557	BL36	BL-cell-line	North Africa	ND
**782**	LN827548	P3HR1_c16	BL-cell-line	Nigeria	ND
**783**	LN827800	Jijoye	BL-cell-line	Nigeria	ND
**784**	LN827556	Cheptages	BL-cell-line	Africa	ND
**785**	DQ279927	AG876	BL	Ghana	ND
**786**	NC_009334	AG876	BL-cell-line	Ghana	ND
**787**	LN827544	Wewak_2	BL-cell-line	Papua New Guinea	ND
**788**	MH883770	ebv30	HSOT	United Kingdom	ND
**789**	MH883769	ebv27	HSOT	United Kingdom	ND
**790**	MH883768	ebv25	HSOT	United Kingdom	ND
**791**	MH883766	ebv22	HSOT	United Kingdom	ND
**792**	MH883765	ebv21	HSOT	United Kingdom	ND
**793**	MH883764	ebv19	HSOT	United Kingdom	ND
**794**	MH883763	ebv17	HSOT	United Kingdom	ND
**795**	MH883761	ebv15	HSOT	United Kingdom	ND
**796**	MH883759	ebv13	HSOT	United Kingdom	ND
**797**	MH883758	ebv9	HSOT	United Kingdom	ND
**798**	MH883757	ebv8	HSOT	United Kingdom	ND
**799**	MH883756	ebv7	HSOT	United Kingdom	ND
**800**	MH883755	ebv6	HSOT	United Kingdom	ND
**801**	LS992277	EBV17	HSOT	United Kingdom	ND
**802**	LS992264	P11-871	IM	Japan	ND
**803**	LS992261	P1-812	IM	Japan	ND
**804**	MF547492	E1577_BCv7	ND	USA	ND
**805**	MF547491	E1577_OWv7	IM	USA	ND
**806**	AP015015	SNU-719	GC-cell-line	Korea	ND
**807**	LN827595	sLCL-IS1.03	sLCL-PTDL	Australia	ND
**808**	LN827594	sLCL-IS1.07	sLCL-PTDL	Australia	ND
**809**	LN827593	sLCL-IS1.12	sLCL-PTDL	Australia	ND
**810**	LN827592	sLCL-IS1.10	sLCL-PTDL	Australia	ND
**811**	LN827590	sLCL-IM1.05	sLCL-IM	Australia	ND
**812**	LN827585	sLCL-1.04	sLCL	Kenya	ND
**813**	LN827584	sLCL-IS1.06	sLCL-PTDL	Australia	ND
**814**	LN827583	sLCL-IM1.17	sLCL-IM	Australia	ND
**815**	LN827582	sLCL-BL1.03	sLCL	Kenya	ND
**816**	LN827581	sLCL-1.05	sLCL	Kenya	ND

**817**	LN827579	sLCL-1.13	sLCL	Kenya	ND
**818**	LN827577	sLCL-1.17	sLCL	Kenya	ND
**819**	LN827576	sLCL-IS1.20	sLCL-PTDL	Australia	ND
**820**	LN827575	sLCL-IS1.14	sLCL-PTDL	Australia	ND
**821**	LN827574	sLCL-1.09	sLCL	Kenya	ND
**822**	LN827573	sLCL-1.10	sLCL	Kenya	ND
**823**	LN827572	sLCL-IS1.18	sLCL-PTDL	Australia	ND
**824**	LN827571	sLCL-BL1.20	sLCL	Kenya	ND
**825**	LN827570	sLCL-IS1.01	sLCL-PTDL	Australia	ND
**826**	LN827569	sLCL-IS1.11	sLCL-PTDL	Australia	ND
**827**	LN827568	sLCL-1.24	sLCL	Kenya	ND
**828**	LN827567	sLCL-IM1.09	sLCL-IM	Australia	ND
**829**	LN827566	sLCL-1.06	sLCL	Kenya	ND
**830**	LN827565	sLCL-1.07	sLCL	Kenya	ND
**831**	LN827564	HL04	HL	United Kingdom	ND
**832**	LN827562	sLCL-1.19	sLCL	Kenya	ND
**833**	LN827559	pLCL-TRL595	sLCL-PTDL	USA	ND
**834**	LN827558	sLCL-1.02	sLCL	Kenya	ND
**835**	LN827553	sLCL-IS1.08	sLCL-PTDL	Australia	ND
**836**	LN827552	sLCL-1.08	sLCL	Kenya	ND
**837**	LN827551	Makau	BL	Kenya	ND
**838**	LN827550	sLCL-1.11	sLCL	Kenya	ND
**839**	LN827546	HL02	HL	United Kingdom	ND
**840**	LN827524	HL11	HL	United Kingdom	ND
**841**	LN827523	L591	HL	Germany	ND
**842**	LN827522	HL09	HL	United Kingdom	ND
**843**	LN824226	HL01	HL	United Kingdom	ND
**844**	LN824225	HL08	HL	United Kingdom	ND
**845**	LN824207	pLCL-TRL1-pre	sLCL-PTDL	USA	ND
**846**	LN824206	pLCL-TRL1-post	sLCL-PTDL	USA	ND
**847**	LN824204	HL05	HL	United Kingdom	ND
**848**	LN824203	Mak_1	BL-cell-line	Kenya	ND
**849**	LN827545	Daudi	BL	Kenya	ND
**850**	LN827527	M-ABA	LCL-NPC	Africa	ND
**851**	LN827526	BL37	BL-cell-line	Africa	ND
**852**	LN827578	sLCL-IS1.13	sLCL-PTDL	Australia	ND
**853**	LN827739	LCL_B958_delEber2	LCL	USA	ND
**854**	LN827597	sLCL-IS1.04	sLCL-PTDL	Australia	ND
**855**	LN827596	sLCL-IM1.02	sLCL-IM	Australia	ND
**856**	LN827588	sLCL-IS1.19	sLCL-PTDL	Australia	ND
**857**	LN824208	Akata	BL	Japan	ND
**858**	LS992276	EBV8	HSOT	United Kingdom	ND
**859**	LS992269	P12-1026	IM	Japan	ND
**860**	LS992268	P6-1751	IM	Japan	ND
**861**	LN827555	X50-7	cell-line	USA	ND
**862**	MH883785	P11-871	IM	Japan	ND
**863**	MH883767	ebv23	HSOT	United Kingdom	ND
**864**	MH590444	HKHD75	HS	China	ND
**865**	MH590501	HKHD132	HS	China	ND

**866**	MH590496	HKHD127	HS	China	ND
**867**	MH590495	HKHD126	HS	China	ND
**868**	MH590494	HKHD125	HS	China	ND
**869**	MH590493	HKHD124	HS	China	ND
**870**	MH590491	HKHD122	HS	China	ND
**871**	MH590490	HKHD121	HS	China	ND
**872**	MH590489	HKHD120	HS	China	ND
**873**	MH590487	HKHD118	HS	China	ND
**874**	MH590486	HKHD117	HS	China	ND
**875**	MH590485	HKHD116	HS	China	ND
**876**	MH590483	HKHD114	HS	China	ND
**877**	MH590481	HKHD112	HS	China	ND
**878**	MH590478	HKHD109	HS	China	ND
**879**	MH590477	HKHD108	HS	China	ND
**880**	MH590476	HKHD107	HS	China	ND
**881**	MH590475	HKHD106	HS	China	ND
**882**	MH590472	HKHD103	HS	China	ND
**883**	MH590468	HKHD99	HS	China	ND
**884**	MH590465	HKHD96	HS	China	ND
**885**	MH590464	HKHD95	HS	China	ND
**886**	MH590462	HKHD93	HS	China	ND
**887**	MH590461	HKHD92	HS	China	ND
**888**	MH590460	HKHD91	HS	China	ND
**889**	MH590459	HKHD90	HS	China	ND
**890**	MH590455	HKHD86	HS	China	ND
**891**	MH590451	HKHD82	HS	China	ND
**892**	MH590450	HKHD81	HS	China	ND
**893**	MH590449	HKHD80	HS	China	ND
**894**	MH590445	HKHD76	HS	China	ND
**895**	MH590443	HKHD74	HS	China	ND
**896**	MH590440	HKHD71	HS	China	ND
**897**	MH590439	HKHD70	HS	China	ND
**898**	MH590438	HKHD69	HS	China	ND
**899**	MH590437	HKHD68	HS	China	ND
**900**	MH590435	HKHD66	HS	China	ND
**901**	MH590432	HKHD63	HS	China	ND
**902**	MH590431	HKHD62	HS	China	ND
**903**	MH590430	HKHD61	HS	China	ND
**904**	MH590429	HKHD60	HS	China	ND
**905**	MH590428	HKHD59	HS	China	ND
**906**	MH590427	HKHD58	HS	China	ND
**907**	MH590424	HKHD55	HS	China	ND
**908**	MH590419	HKHD50	HS	China	ND
**909**	MH590413	HKHD44	HS	China	ND
**910**	MH590412	HKHD43	HS	China	ND
**911**	MH590411	HKHD42	HS	China	ND
**912**	MH590405	HKHD36	HS	China	ND
**913**	MH590404	HKHD35	HS	China	ND
**914**	MH590401	HKHD32	HS	China	ND

**915**	MH590400	HKHD31	HS	China	ND
**916**	MH590397	HKHD28	HS	China	ND
**917**	MH590396	HKHD27	HS	China	ND
**918**	MH590394	HKHD25	HS	China	ND
**919**	MH590393	HKHD24	HS	China	ND
**920**	MH590392	HKHD23	HS	China	ND
**921**	MH590391	HKHD22	HS	China	ND
**922**	MH590390	HKHD21	HS	China	ND
**923**	MH590388	HKHD19	HS	China	ND
**924**	MH590385	HKHD16	HS	China	ND
**925**	MH590384	HKHD15	HS	China	ND
**926**	MH590383	HKHD14	HS	China	ND
**927**	MH590382	HKHD13	HS	China	ND
**928**	MH590380	HKHD11	HS	China	ND
**929**	MH590376	HKHD7	HS	China	ND
**930**	MH590374	HKHD5	HS	China	ND
**931**	MH590373	HKHD4	HS	China	ND
**932**	LN827799	sLCL-IM1.16	sLCL-IM	Australia	ND
**933**	LN827586	sLCL-IS1.15	sLCL-PTDL	Australia	ND
**934**	LN827561	YCCEL1	GC-cell-line	South Korea	ND
**935**	LN827549	D3201.2	NPC	China	ND
**936**	LN827547	HKN15	sLCL	China	ND
**937**	LN824224	HKN19	NPCsLCL	China	ND
**938**	LN824209	HKN14	NPC	China	ND
**939**	LN824205	sLCL-1.12	sLCL	Kenya	ND
**940**	LN824142	Saliva1_assembly	HS	United Kingdom	ND
**941**	MH590378	HKHD9	HS	China	ND
**942**	MH590537	HKNPC26	NPC	China	ND
**943**	MH590425	HKHD56	HS	China	ND
**944**	MH144223	NKTCL-SG12	NKTCL	Singapore	ND
**945**	MH144222	NKTCL-SG11	NKTCL	Singapore	ND
**946**	MH144220	NKTCL-SG09	NKTCL	Singapore	ND
**947**	MH144215	NKTCL-SG04	NKTCL	Singapore	ND
**948**	MH144213	NKTCL-SG02	NKTCL	Singapore	ND
**949**	MH144212	NKTCL-SG01	NKTCL	Singapore	ND
**950**	MH590572	HKNPC61	NPC	China	ND
**951**	MH590567	HKNPC56	NPC	China	ND
**952**	MH590563	HKNPC52	NPC	China	ND
**953**	MH590562	HKNPC51	NPC	China	ND
**954**	MH590561	HKNPC50	NPC	China	ND
**955**	MH590558	HKNPC47	NPC	China	ND
**956**	MH590550	HKNPC39	NPC	China	ND
**957**	MH590549	HKNPC38	NPC	China	ND
**958**	MH590547	HKNPC36	NPC	China	ND
**959**	MH590546	HKNPC35	NPC	China	ND
**960**	MH590544	HKNPC33	NPC	China	ND
**961**	MH590539	HKNPC28	NPC	China	ND
**962**	MH590533	HKNPC22	NPC	China	ND
**963**	MH590530	HKNPC19	NPC	China	ND

**964**	MH590529	HKNPC18	NPC	China	ND
**965**	MH590526	HKNPC15	NPC	China	ND
**966**	MH590518	HKNPC7	NPC	China	ND
**967**	MH590517	HKNPC6	NPC	China	ND
**968**	MH590511	HKHD142	HS	China	ND
**969**	MH590506	HKHD137	HS	China	ND
**970**	MH590458	HKHD89	HS	China	ND
**971**	MH144216	NKTCL-SG05	NKTCL	Singapore	ND
**972**	MH590510	HKHD141	HS	China	ND
**973**	MH590508	HKHD139	HS	China	ND
**974**	MH590488	HKHD119	HS	China	ND
**975**	MH590473	HKHD104	HS	China	ND
**976**	MH590467	HKHD98	HS	China	ND
**977**	MH590466	HKHD97	HS	China	ND
**978**	MH590448	HKHD79	HS	China	ND
**979**	MH590446	HKHD77	HS	China	ND
**980**	MH590441	HKHD72	HS	China	ND
**981**	MH590436	HKHD67	HS	China	ND
**982**	MH590421	HKHD52	HS	China	ND
**983**	MH590410	HKHD41	HS	China	ND
**984**	MH590381	HKHD12	HS	China	ND
**985**	MH590398	HKHD29	HS	China	ND
**986**	MH590503	HKHD134	HS	China	ND
**987**	MH590492	HKHD123	HS	China	ND
**988**	MH590417	HKHD48	HS	China	ND
**989**	LN831023	sLCL-2.22	sLCL	Kenya	ND
**990**	LN827563	sLCL-1.18	sLCL	Kenya	ND
**991**	LN827591	sLCL-2.15	sLCL	Kenya	ND
**992**	LN827589	sLCL-IS2.01	sLCL-PTDL	Australia	ND
**993**	LN827587	sLCL-2.21	sLCL	Kenya	ND
**994**	LN827580	sLCL-2.16	sLCL	Kenya	ND
**995**	LN827560	sLCL-2.14	sLCL	Kenya	ND
**996**	MH883784	P9-2631	IM	Japan	ND
**997**	MH883783	P8-414	IM	Japan	ND
**998**	MH883780	P5-1294	IM	Japan	ND
**999**	MH883778	P4-2274	IM	Japan	ND
**1000**	MH883776	P3-2670	IM	Japan	ND
**1001**	MH883774	P2-1213	IM	Japan	ND
**1002**	LS992266	P8-414	IM	Japan	ND
**1003**	LS992265	P9-2631	IM	Japan	ND
**1004**	LS992263	P5-1294	IM	Japan	ND
**1005**	LS992260	P3-2670	IM	Japan	ND
**1006**	LS992259	P2-1213	IM	Japan	ND

Angioimmunoblastic T lymphoma (AIL), Aggressive NK-cell leukemia (ANCL), AIDS-related lymphoma (ARL), Burkitt lymphoma (BL), Chronic active EBV infection (CAEBV), Diffuse large B-cell lymphoma (DLBCL), Gastric Cancer (GC), Heath-SolidOrganTransplant (HSOT), Hodgkin lymphoma (HL), Health-Saliva (HS), Infectious mononucleosis (IM), Lung Carcinoma (LC), Lymphoblastoid cell line (LCL), Lymphoepithelioma (LE), Lymphoid malignancy (LM), Natural killer T-cell lymphoma (NKTCL), Nasopharyngeal Carcinoma (NPC), Post-transplant B lymphoma (PTBL), Posttransplant lymphoproliferative disorder (PTLD), Spontaneous lymphoblastoid cell line (sLCL), and Not determined (ND).

Supplementary Table S2 List of sequences of each putative recombinant event identified by RDP4 and breakpoint prediction.

Event	Sequence names	Start	End
**1 (1)**	**NPCT021, NPCT049**	**661**	**1508**
**2 (4)**	AH_Saliva_8489, HKHD6, HKHD7, HKHD9, HKHD13, HKHD19, HKHD27, HKHD28, HKHD31, HKHD32, HKHD42, HKHD44, HKHD50, HKHD55, HKHD56, HKHD59, HKHD60, HKHD61, HKHD66, HKHD70, HKHD71, HKHD74, HKHD75,HKHD82, HKHD86, HKHD89, HKHD99, HKHD108, HKHD112, HKHD114, HKHD124, HKHD126, HKHD132, HKHD142, HKN14, HKNPC6, HKNPC7, HKNPC19, HKNPC26, HKNPC28, HKNPC35, HKNPC47, HKNPC52, HKNPC56, HKNPC61, HKTCL-SC10, HKTCL-SC11, HLT010, HS15, HS011, HS032, HS033, HS035, HS036, HS039, IMS_Saliva_120, IMS_Saliva_204, NKTCL-SG07, NKTLT007, NNPCT004, NPCT090, NPCT065, NPCT060, NPCS052	2807	1421
**3 (6)**	GK_LY91_BWA	1483	1614
**4 (7)**	GC-EBV2, GDGC2, HKNPC60, HS012, UPN310_CD56+, UPN366_CD3+	2790	1314
**5 (8)**	HKNPC45	2262	2796
**6 (9)**	HKNPC39	599	1086
**7 (10)**	HKHD43, HKNPC39, NKTCL-SC01, UPN431_CD3+, UPN431_CD19+, UPN431_CD56+	1977	2449
**8 (12)**	AH_Saliva_8471, AH_Saliva_8489, HKHD7, HKHD9, HKHD13, HKHD19, HKHD27, HKHD28, HKHD31, HKHD32, HKHD42, HKHD44, HKHD50, HKHD55, HKHD56, HKHD60, HKHD61, HKHD66, HKHD70, HKHD71, HKHD74, HKHD75, HKHD82, HKHD86, HKHD89, HKHD99, HKHD108, HKHD112, HKHD114, HKHD124, HKHD126, HKHD132, HKN14, HKNPC6, HKNPC7, HKNPC19, HKNPC26, HKNPC28, HKNPC47, HKNPC52, HKNPC56, HKNPC61, HKTCL-SC10, HS032, HLT010, HS033, HS035, HS036, NKTCL-SG07, NPCT090, NKLT007, NKTCL-SC11	Unknown	Unknown

Supplementary Table S3 RDP4 methods with supporting p-values for putative recombinant events in the EBNA3A gene.

Event (n)	RDP	GENCONV	MaxChi	Chimaera	SciScan	3Seq
1 (1)	2.431×10^{-31}	4.862×10^{-24}	1.857×10^{-20}	9.807×10^{-21}	1.016×10^{-25}	6.081×10^{-57}
2 (4)	1.559×10^{-2}	2.622×10^{-4}	1.020×10^{-4}	1.491×10^{-4}	3.327×10^{-6}	4.828×10^{-9}
3 (6)	3.699×10^{-7}	1.857×10^{-4}	-	-	-	-
4 (7)	10000	8.177×10^{-3}	5.572×10^{-4}	7.608×10^{-3}	8.673×10^{-4}	1.455×10^{-3}
5 (8)	7.515×10^{-3}	-	-	-	-	5.716×10^{-6}
6 (9)	2.009×10^{-3}	-	1.286×10^{-2}	9.606×10^{-3}	2.041×10^{-3}	-
7 (10)	3.759×10^{-3}	1.208×10^{-2}	-	-	2.596×10^{-2}	3.538×10^{-3}
8 (12)	4.302×10^{-1}	-	-	2.368×10^{-2}	5.801×10^{-3}	1.100×10^{-2}

All values expressed for methods are the p-values with Bonferroni correction

Supplementary Table S4: Characteristics of 24 South American EBVs sequences.

Country	Nº of sequences	EBV-1	EBV-2	Disease	Collection year (nº of sequences)
**Argentina**	6	5	1	BL	1980-1981 (4)<br>2015 (2)
**Brazil**	6	6	0	BL	1980 (all)
**Chile**	12	9	3	GC	2008 (2), 2009 (8) and 2011 (2)

BL Burkitt Lymphoma, GC Gastric Cancer.

Supplementary Table S5: EBNA3A canonical and non-canonical NLS amino acid (aa) positions and identity of the EBV types:

NLS	EBV-1	EBV-2	Nº of aa	Start	End	Identity between EBV-1 and EBV-2 (%)
**1**	KRKR	KRKR	4	63	66	100.0
**2**	RDRRRNPASR	RDRRRNPASR	10	146	155	100.0
**3**	PKVKRPP	TKHRRPP	7	375	381	57.1
**4**	RAGKR	RAGKG	5	394	398	80.0
**5**	RRARER	KRTRER	6	573	578	66.7
**6**	RDKLAR	RDKLAR	6	598	603	100.0

Amino acids found to have mutations are underlined.

Supplementary Table S6 EBNA3A amino acid (aa) deletion patterns.

Deletion	EBV-1	EBV-2	Amino acid
**1**	M	–	15
**8**	LAAQGMAY	P	451-458
**5**	PPVSP	L	480-484
**5´**	PVYPK	M	548-552

The deletion numbers represent the number of amino acids deleted. Gap (–) indicates a complete deletion.

Supplementary Table S7: Putative recombination regions identified by Gubbins.

Recombinant block	Start	End
**I**	171	403
**II**	487	610
**III**	**720**	**1485**
**IV**	**1320**	**1451**
**V**	1552	1725
